# Traditional Chinese medicine for smoking cessation: An umbrella review of systematic reviews and meta-analysis of randomized controlled trials

**DOI:** 10.18332/tid/174090

**Published:** 2023-11-15

**Authors:** Chun-Li Lu, Xin-Yan Jin, Qian-Yun Wang, Xiao-Ying Chen, Ruo-Xiang Zheng, Chao-Jie Wang, Jing-Jing Jiang, Shu-Yu Qiao, Si-Hong Yang, Wei-Han Zhang, Si-Yi Chen, Jia-Xuan Li, Xue-Han Liu, Yu-Si Suo, Jian-Hua Wang, Xue Xue, Li-Rong Liang, Nicola Robinson, Jian-Ping Liu

**Affiliations:** 1 Centre for Evidence-Based Chinese Medicine, Beijing University of Chinese Medicine, Beijing, China; 2 Dongzhimen Hospital, Beijing University of Chinese Medicine, Beijing, China; 3 Department of the Graduate School, Heilongjiang University of Chinese Medicine, Harbin, China; 4 Graduate Institute of Interpretation and Translation, Shanghai International Studies University, Shanghai, China; 5 Center of Evidence-Based Chinese Medicine, China Academy of Chinese Medical Sciences, Beijing, China; 6 School of Public Health, Peking University, Beijing, China; 7 School of Qihuang, Chinese Medicine, Beijing University, Beijing, China; 8 Affiliated Hubei Provincial Hospital of Traditional Chinese Medicine, Hubei University of Traditional Chinese Medicine, Wuhan, China; 9 Hubei Key Laboratory of theory and application research of liver and kidney in traditional Chinese medicine, Wuhan, China; 10 School of Traditional Chinese Medicine, Liaoning University of Traditional Chinese Medicine, Liaoning, China; 11 Department of Research on Tobacco Dependence Therapies, Beijing Institute of Respiratory Medicine and Beijing Chao-Yang Hospital, Capital Medical University, Beijing, China; 12 Institute of Health and Social Care, London South Bank University, London, United Kingdom

**Keywords:** smoking cessation, traditional Chinese medicine, systematic review, acupoint stimulation, tobacco withdrawal syndrome

## Abstract

**INTRODUCTION:**

Traditional Chinese medicine (TCM) may have special advantages in facilitating smoking cessation, but consensus on effectiveness is lacking. We aim to comprehensively review, update, and refine current evidence on TCM effectiveness and safety.

**METHODS:**

Nine databases were searched from their inception up to 28 February 2023. Systematic reviews (SRs) and meta-analysis of TCM for smoking cessation were identified and retrieved. Additional databases and hand searches of RCTs from included SRs were performed for data pooling. Cochrane ROB tools and AMSTAR-2 were used to evaluate the methodological quality of RCTs and SRs, respectively. RCT data are presented as relative risks (RR) or mean differences (MD) with 95% confidence intervals (CI) using RevMan 5.4.

**RESULTS:**

Thirteen SRs involving 265 studies with 33081 participants were included. Among these 265 studies, 157 were duplicates (58.36%) and 52 were non-RCTs (19.62%). Combined with the remaining 56 RCTs identified through hand searches, 88 RCTs involving 12434 participants were finally included for data synthesis. All the SRs focused on acupoint stimulation, and the majority were of low or very low quality. The methodological quality of RCTs was either unclear or high risk. For continuous abstinence rate, TCM external interventions were better than placebo in 6 months to 1 year (RR=1.60; 95% CI: 1.14–2.25; I^2^=27%; n=5533 participants). Compared with placebo, TCM external application was effective in reducing nicotine withdrawal symptoms, and the effect was gradually stable and obvious in the fourth week (MD= -4.46; 95% CI: -5.43 – -3.49; n=165 participants). Twelve RCTs reported adverse events as outcome indicators for safety evaluation, and no serious adverse events occurred.

**CONCLUSIONS:**

Despite the methodological limitations of the original studies, our review suggests that TCM intervention shows potential effectiveness on the continuous abstinence rate. Extending the intervention time can enhance the effect of TCM on nicotine withdrawal symptoms. Referred to adverse events, more data for safety evaluation are required.

## INTRODUCTION

Smoking is a global public health problem, contributing to approximately six million global annual deaths^1^. Smoking is a risk factor associated with various diseases (e.g. respiratory, cardio-cerebrovascular diseases, diabetes, male sexual dysfunction)^2^. Therefore, achieving smoking cessation is an important and cost-effective intervention^3^. Non-pharmacological smoking cessation treatment includes behavioral interventions such as counseling or education to help motivate patients to quit smoking. The more mature and recommended interventions are the 5Rs method (Relevance, Risk, Rewards, Roadblocks, Repetition) and the 5As method (Ask, Advise, Assess, Assist, Arrange)^4^. These behavioral interventions are affected by many factors in clinical implementation, such as the experience of physicians, specific implementation methods or smoking cessation settings. Pharmacotherapy is divided into nicotine replacement therapy (NRT) and non-nicotine replacement therapy (non-NRT)^5^. NRT can release nicotine through patches, chewing gum, nasal sprays, inhalants, or other ways to provide a brief nicotine stimulus, thereby relieving or alleviating nicotine withdrawal symptoms. NRT often causes unintended discomfort at the administration site, even non-ischemic chest pain and palpitations^6^. Non-NRT is defined as drugs not containing components of nicotine, bupropion and varenicline are most commonly used. The mechanism of non-NRT is to block nicotinic receptor binding to stop smoking and to relieve symptoms of anxiety and depression associated with smoking cessation. Bupropion and varenicline are widely used and recommended by international guidelines^4,5^. Nausea, dry mouth, and gastrointestinal discomfort caused by varenicline and bupropion are the most common adverse effects, though varenicline is possibly associated with suicidal tendencies^7^.

Traditional Chinese medicine (TCM) tackles smoking cessation using different therapeutic modalities, including acupoint stimulation, acupressure, and herbal medication. A previous study^8^ has shown there is an increasing need for TCM for smoking cessation, but a lack of high-quality evidence remains. Only one Cochrane systematic review^9^ identified the potential use of acupuncture for smoking cessation and smoking cessation-related symptoms.

Considering several systematic reviews (SRs) focusing on a single modality of TCM for smoking cessation, we conducted an umbrella review of SRs and we performed a meta-analysis of randomized controlled trials (RCTs)^10^ to comprehensively review, update, and refine current evidence to confirm TCM effectiveness and safety^11^.

## METHODS

This study followed the methodological process of the Joanna Briggs Institute for an ‘umbrella review’^12^ (a re-evaluation of SRs)^13^. Compared with typical SRs, an umbrella review can synthesize evidence at a higher-level and better identify uncertainties, biases, and knowledge gaps^14,15^. This study was registered on INPLASY (202190001)^16^, and reported following PRISMA-2020^17^.

### Selection criteria

All the systematic reviews and meta-analysis of RCTs were included. Smokers had no restrictions on physical condition, age, occupation, or gender. TCM interventions included oral administration of TCM prescription, TCM external application or acupoint stimulation (acupuncture, electroacupuncture, auricular acupressure, ear acupuncture, catgut-embedding therapy or other acupoint stimulation). Other kinds of treatments for smoking cessation included no treatment group, placebo, nicotine replacement therapy, non-nicotine replacement therapy, or other non-drug therapy.

### Outcomes

Primary outcomes were abstinence rate, which could be divided into continuous abstinence, prolonged abstinence, prolonged abstinence with relapses, and repeated point-prevalence abstinence. Publications that reported total abstinence without classification were regarded as continued abstinence for the follow-up period stated. Secondary outcomes were nicotine withdrawal symptoms, evaluated by the Minnesota Nicotine Withdrawal Scale (MNWS)^18^ and the Fagerstrom test for nicotine dependence (FTND)^19^, and relapse rate. Safe outcomes referred to adverse events resulting from trial participation (other adverse events besides withdrawal symptoms reported in the trials were also extracted).

### Information sources and search strategy

We searched the China National Knowledge Infrastructure (CNKI), WANFANG, CQVIP, SINOMED, PubMed, Embase, and Cochrane Library of SRs and meta-analysis, from their inception to 28 February 2023. For more details on the search strategies see Supplementary file Table 1.

Considering the data analysis, in addition to the databases mentioned above, we also conducted an additional search of RCTs from Clinicaltrials.gov and the Chinese Clinical Trial Registry.

### Study selection and data collection

For SRs, after removing any duplicates, two authors independently screened the titles and abstracts. Eligibility assessments were performed on the full texts to determine whether to include the study. A third author was invited to adjudicate if there was no agreement.

For RCTs, we performed hand searching from included SRs and systematically searched from the above nine databases and websites. Then we merged the two parts to exclude overlaps.

A pre-designed data extraction form was developed using Excel. After the data extraction was independently conducted by one of the authors, the extracted data were double-checked by another author. The extracted items included: 1) Basic information such as title, authors, publication year, country, sample size, etc.; 2) For SRs, we also extracted other information, such as characteristics of the included participants, details of the intervention(s), comparator(s), and quality assessment; and 3) For RCTs, we added extraction of the efficacy and safety data.

### Quality assessment

*Systematic reviews*

The AMSTAR-2 tool^20^ was used to evaluate the methodological quality of the included SRs by two authors. AMSTAR-2 contained 16 items and seven of which were critical. Each of the items had three options: ‘Yes’, ‘Partial Yes’, ‘No’, and some items additional with ‘No meta-analysis’. The overall study quality was evaluated on a comprehensive scale of high, medium, low, or very low, according to the main items.

*Randomized controlled trials*

The methodological quality was assessed independently by two authors using the Cochrane risk of bias tool (Cochrane ROB Tool)^21^. The random sequence generation, allocation concealment, blinding of participants and personnel, blinding of outcome assessment, incomplete outcome data, selective reporting, and other bias (funding and conflicts of interest) of the included trials were judged as ‘low risk’, ‘high risk’, or ‘unclear’. Any disagreements were resolved by discussion with a third author.

### Data analysis

In terms of SRs, we did not perform data synthesis directly to avoid data supposition but used a qualitative approach to present a summary of the findings.

The extracted data from RCTs for meta-analysis of effect sizes were analyzed by Revman 5.4.0. We used mean difference (MD) with a 95% confidence interval (CI) for continuous data, and relative risk (RR) with a 95% CI for dichotomous data. Subgroup analysis based on route of administration (such as internal or external) or different TCM interventions (such as acupoint stimulation or Chinese herbal preparations) was performed, where possible. Publication bias was evaluated with funnel plots when more than ten trials were included in a meta-analysis. As recommended by the Cochrane Handbook for Systematic Reviews of Interventions, I² was used to test the statistical heterogeneity^21^. The fixed effect model was applied when the included trials were considered to have low heterogeneity (I^2^ <30%) or mild heterogeneity (30%< I^2^ <75%). For the data with high heterogeneity (I^2^ >75%), we used random effects model to pool. A p<0.05 indicated that there was a statistical difference. Where possible, sensitivity analyses were performed by excluding low-quality literature or changing statistical models to determine the robustness of the conclusions.

## RESULTS

### Study selection

**Figure 1:**
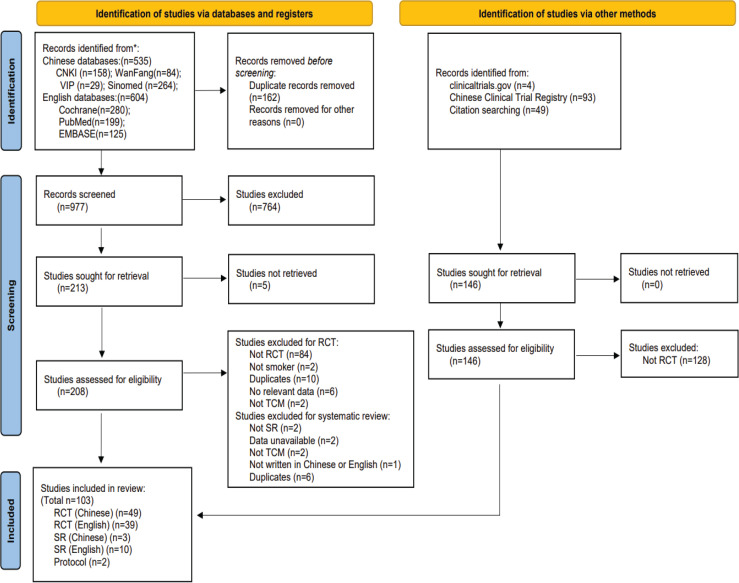
Flow diagram of study retrieval

A total of 1139 studies were retrieved from Chinese and English databases, and thirteen SRs^9,22,23^ were finally included. A total of 88 RCTs (Supplementary file Table 2) were used for data synthesis, and we also searched two RCT protocols without study results (Supplementary file Table 3). The flow diagram of study selection is shown in Figure 1.

### Study characteristics

*Systematic reviews*

**Figure 2:**
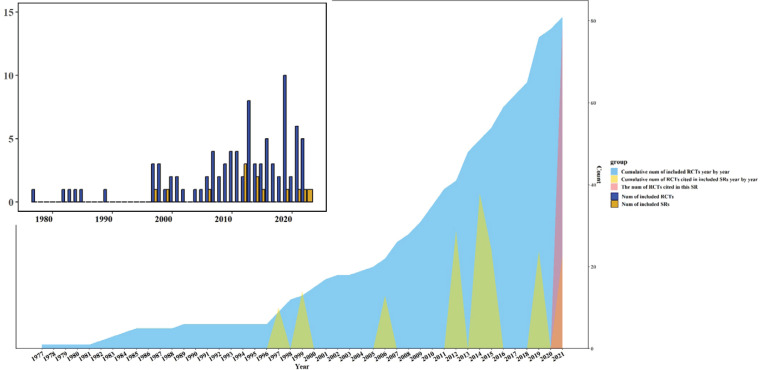
Distribution of RCTs and SRs on TCM on smoking cessation

Thirteen SRs involving 265 studies with 33801 subjects were finally included. Among the 265 studies, 157 were duplicates (58.36%) and 52 were non-RCTs of TCM smoking cessation (19.62%). There was no increase year by year or explosive growth in a certain year in SRs, while the publication of RCTs showed an increasing trend year by year. We counted the number of SRs and RCTs published each year and tried to show the relationship between them (Figure 2). All the SRs’ topics were focused on the effects of acupoint stimulation, including acupuncture, electroacupuncture, auricular acupressure, and laser acupuncture, without oral TCM treatments or TCM external application. The main outcome was the continuous abstinence rate, but it lacked specific definitions and measurement times. In conclusion, included SRs indicated that acupoint stimulation was potentially effective or effective on abstinence rate. Regarding quality assessment, most SRs (8 studies, 61.54%) used the Cochrane ROB tool to assess the quality of the included RCTs. Details of the characteristics are shown in Table 1 and in Supplementary file Table 4.

**Table 1:** Summary of characteristics and evaluation results of included systematic reviews22-33 of TCM for smoking cessation (N=13)

*Authors Year*	*Language*	*Included trials*	*Included participants*	*Study population (Total: M/F)*	*Age (years)*	*Combined with other diseases*	*Quality assessment methods*	*AMSTAR-2*
Ashenden et al.^22^ 1997	English	10	2707	NR	NR	No	NR	Very low
Cheng et al.^23^ 2012	English	20	4923	NR	16–73	No	Jadad score	Low
Di et al.^24^ 2014	English	25	3735	NR	NR	No	Cochrane risk of bias	Low
Wang et al.^25^ 2019	English	24	3984	1157/627	23–85	No	Cochrane risk of bias	Low
Kim et al.^26^ 2012	English	29	NR	NR	NR	No	Cochrane risk of bias	Very low
Dai et al.^27^ 2021	English	23	2706	NR	20–71.2	NR	Cochrane risk of bias	Low
Tahiri et al.^28^ 2012	English	6	823	NR	37.5–53.7	NR	Cochrane risk of bias	Very low
White et al.^9^ 2014	English	38	2868	NR	12–72	NR	Cochrane risk of bias	Medium
White and Moody^29^ 2006	English	13	1345	NR	NR	NR	NR	Very low
Liu et al.^30^ 2015	Simplified Chinese	24	3084	NR	NR	NR	Self-designed	Very low
White et al.^31^ 1999	English	14	3486	NR	NR	NR	Self-defined	Very low
Liu et al.^32^ 2023	Simplified Chinese	23	2164	NR	NR	NR	Cochrane risk of bias	Very low
Kuang et al.^33^ 2022	Simplified Chinese	16	1976	NR	NR	NR	Cochrane risk of bias	Very low

TCM: Traditional Chinese medicine. NR: Not reported. M: male. F: female. AMSTAR-2: A measure tool to assess systematic reviews.

*Randomized controlled trials*

Eighty-eight RCTs through database search and hand search were finally included for data synthesis. Most RCTs (50 studies, 56.82%) were published in China, and more than half of them (64 studies, 72.73%) had sample sizes ranging from 31 to 150 participants.

**Figure 3:**
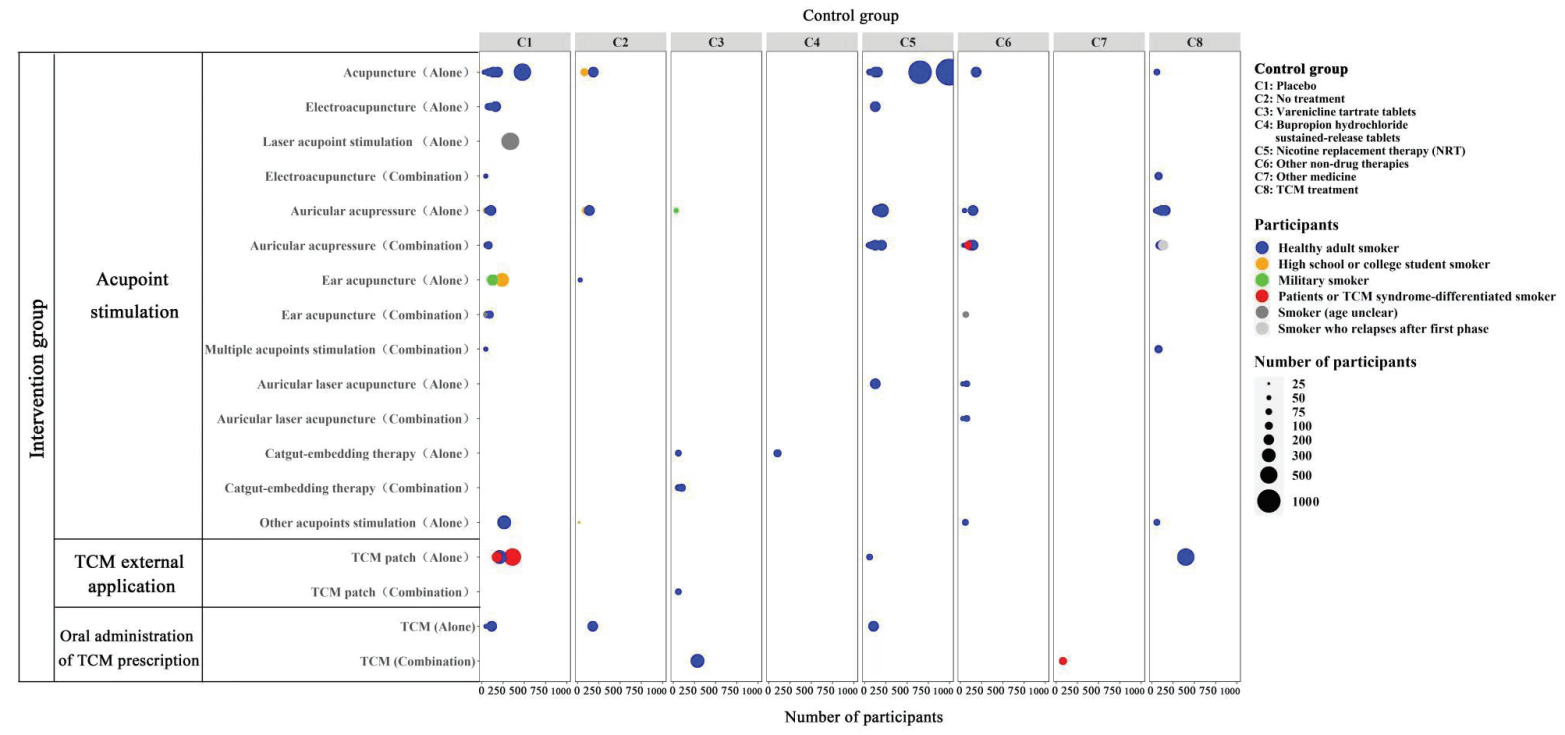
Bubble chart of included RCTs of TCM for smoking cessation (N=88)

The majority of RCTs (81 studies, 92.05%) included healthy adult smokers. Acupoint stimulation was the most common intervention (82 studies, 93.18%), and a small number of studies focused on oral administration of TCM prescription and TCM external application. Control groups involved placebo, no treatment, positive medicines (varenicline and bupropion), NRT products, non-drug therapies, other medicine (ceftriaxone), and TCM treatments. The primary outcomes were still abstinence rates (Supplementary file Table 5). A bubble chart of participants, interventions, and controls from the 88 RCTs provides more details in Figure 3.

### Quality assessment

*Systematic reviews*

By assessing the quality of 13 SRs, we used the AMSTAR-2 tool and found all were of low or very low quality except for one Cochrane review^9^ (Table 1).

*Randomized controlled trials*

Overall, the quality of the included 88 RCTs was low. In all, 55 studies (62.5%) had a high risk of bias in the blinding of participants and personnel, and there was much unreported information in other aspects such as random sequence generation, allocation concealment, and selective reporting. The risk of bias assessment is shown in Supplementary file Figure 1.

*Random sequence generation*

Forty-six studies (52.27%) were assessed as ‘low risk’ and used random number tables or central randomization. The others were ‘unclear’ or ‘high risk’ for using possible wrong methods or failing to report specific randomization methods.

*Allocation concealment*

Most studies (n=65; 73.86%) were ‘unclear’, as random concealment was not reported. Among the other 23 studies, 20 reported random concealment using central randomization or opaque sealed envelopes, and were rated as ‘low risk’, while the other studies were ‘high risk’ for their open-label.

*Blinding of participants and personnel*

Fifty-five studies (62.5%) were ‘high risk’ in performance bias, primarily because blinding was broken or incomplete; 24 studies (27.27%) were ‘low risk’ as there were details on the implementation of adequate blinding. The remaining studies were ‘unclear’ because they did not report the use of blinding, or the effect of blinding was uncertain.

*Blinding of outcome assessment*

Fifty-nine studies (67.05%) did not report specific details of detection bias, so they were ‘unclear’; 12 studies (13.64%) were ‘low risk’ with clear blinding details of outcomes or objective outcome measures (Supplementary file Table 6).

*Incomplete outcome data*

Most studies (n=73; 82.95%) had no missing data or the missing data had little effect on the outcome (the dropout rate was <20%), so they were regarded as ‘low risk’. Two studies lost >20% of their data and were therefore considered ‘high risk’ (Supplementary file Table 6).

*Selective reporting*

Fourteen trials (15.91%) with registered trial protocols and registration numbers were ‘low risk’. The outcomes in the two studies were not reported as expected in the methods section, so they were ‘high risk’. The remaining 72 studies were ‘unclear’ because insufficient information was available.

*Other sources of bias*

Fifty-five studies (62.5%) without funding or conflict of interest statements were ‘low risk’ and the others were ‘unclear’.

### Primary outcomes of interventions

Abstinence rates in all included RCTs were divided into point prevalence abstinence rate and continuous abstinence rate according to time point and different measurement methods.

*Point prevalence abstinence rate*

The results of the meta-analysis showed that there was no statistical difference between the effect of TCM and placebo or other conventional medicine (varenicline, bupropion, or NRT) at point prevalence abstinence. However, the effect of TCM intervention was superior to other non-drug interventions in terms of 2-month point prevalence abstinence rate (RR=3.00; 95% CI: 1.29–7.00; n=150 participants) (Supplementary file Table 7).

*Continuous abstinence rate*

For the continuous abstinence rate in seven days or less, TCM intervention was associated with a high continuous abstinence rate compared with placebo (RR=3.00; 95% CI: 0.13–70.16; n=148 participants) or NRT (RR=0.33; 95% CI: 0.01–7.87; n=160 participants).

TCM had a greater advantage in 7 to 30 days continuous abstinence rate compared to the placebo group (RR=1.53; 95% CI: 1.29–1.80, I^2^=54%; n=81031 participants). But there were no significant differences between TCM and NRT (RR=0.75; 95% CI: 0.34–1.64; n=2142 participants).

For continuous abstinence rate in 1 to 6 months, the differences between TCM intervention and placebo were not significant (RR= 1.52; 95% CI: 0.92–2.51, I^2^=57%; n=8710 participants). While the combination of TCM and varenicline was higher than varenicline alone (RR=1.35; 95% CI: 1.15–1.58; n=1286 participants).

As for the continuous abstinence rate in 6 months to 1 year, the effect of TCM was better than placebo (RR=1.60; 95% CI: 1.14–2.25, I^2^=27%; n=5533 participants) and no treatment group (RR=2.27; 95% CI: 1.12–4.58; n=1278 participants). Compared with other non-drug therapies, TCM intervention also had advantages (RR=1.89; 95% CI: 1.01–3.53, I^2^=38%; n=2352 participants). Although the difference between TCM and varenicline was not significant (RR=1.28; 95% CI: 0.48–3.42; n=144 participants), the combination of TCM and varenicline had a greater effect (RR=1.45; 95% CI: 1.23–1.76; n=1286 participants).

Judging from the continuous abstinence rate for more than one year, TCM showed no advantages over placebo (RR=0.77; 95% CI: 0.45–1.33, I^2^=46%; n=3150 participants), but its effect was better than that of no treatment group (RR=1.20; 95% CI: 1.02–1.42; n=1150 participants). The effect results of the individual studies above are shown in Supplementary file Table 6.

### Secondary outcomes of interventions

*Minnesota Nicotine Withdrawal Scale (MNWS)*

Compared with placebo, TCM smoking patches applied externally was effective in reducing nicotine withdrawal symptoms, and the effect were more obvious in the fourth week (MD= -4.46; 95% CI: -5.43 – -3.49; n=165 participants) (Supplementary file Figure 2).

*Fagerström test for nicotine dependence (FTND)*

Both TCM (MD= -0.58; 95% CI: -1.11 – -0.06; I^2^=0%; n=2140 participants) and the combination of TCM with non-drug therapies (MD= -0.86; 95% CI: -1.38 – -0.34; I^2^=0%; n=2140 participants) were effective in reducing the FTND scores compared with non-drug therapies. Compared with placebo, TCM external treatment was able to reduce FTND scores for 1 to 4 weeks, improving over time (Supplementary file Figure 3).

*Relapse rate*

Compared with placebo, TCM can significantly reduce the rate of relapse (RR=0.54; 95% CI: 0.33–0.91; I^2^=36%; n=3228 participants). But there was no significant difference between the TCM and NRT (RR=0.69; 95% CI: 0.27–1.77; I^2^=32%; n=265 participants). The combination of TCM and varenicline had a better effect than varenicline (RR=0.38; 95% CI: 0.16–0.94; n=160 participants).

### Adverse events

Among the 88 RCTs included, twelve studies reported adverse events, among which eleven were external interventions (auricular acupuncture, electroacupuncture, auricular acupressure, acupoint embedding, and smoking cessation patch) and one was through oral administration (Chinese herbal extracts), as shown in Supplementary file Table 8.

### Publication bias and sensitivity analysis

Since no more than ten studies were pooled in each meta-analysis, publication bias analysis of the included studies could not be carried out. We did not perform additional sensitivity analysis because of the substantial clinical heterogeneity associated with the different interventions.

## DISCUSSION

### Summary of findings on systematic reviews for TCM smoking cessation

At the SR level, the most prominent problem was the overlap of included studies. Only 88 RCTs were included in this umbrella review, while the cumulative number of studies included in 13 SRs was as high as 265. The included SRs failed to cover all the current evidence-based evaluation studies on TCM smoking cessation. All the SRs focused on acupuncture or acupoint stimulation, lacking comparisons with other TCM interventions.

Regarding the quality of SRs, except for one published in the Cochrane Library, the other studies were of low or very low quality. The main problems were inadequate reporting and non-standard evidence-based methods. Given the low quality of SRs and the quality of the included RCTs, no definite and clear conclusion could be drawn, and most of them only indicated ‘possibly effective’. It suggests that early registration of systematic reviews should be standardized, and future studies should avoid repetitive work.

### Summary of findings on RCTs for TCM smoking cessation

At present, the most common participants in TCM smoking cessation in RCTs are healthy adult smokers. Fewer studies focus on smoking cessation among teenagers, high school or college students, or people with smoking-related illnesses. Future studies should focus on the possible effectiveness of TCM therapies in these groups.

The most common TCM interventions are acupoint stimulation, including acupuncture, electroacupuncture, auricular acupuncture, and other therapies. A few studies focused on Chinese herbal medicine as interventions, such as smoking cessation tea, applications, or decoctions. In terms of comparisons, sham acupuncture and NRT products are the most common controls. The efficacy of NRT is uncertain, and sham acupuncture is controversial. Hence, further studies should focus on the appropriate design such as positive medicine comparators.

Regarding the outcomes, the abstinence rate is still used as the main indicator, but the measurement time points among the studies were quite different. Criteria for measuring abstinence rates are also not uniform, with the majority measured in the form of patient-report and a lack of validation with physiological measures such as urine cotinine or exhaled CO. For the point prevalence abstinence rate that can reflect the short-term effectiveness of smoking cessation interventions, we suggest that 24 hours might be a suitable observation time point. For the continuous abstinence rate reflecting long-term effectiveness, six months could be used as the observation time point, in accordance with Chinese Clinical Smoking Cessation Guidelines. Apart from abstinence rates, other outcomes such as smoking urge, nicotine dependence, and changes in mood and physical symptoms are also important in reflecting the effectiveness of smoking cessation interventions. We suggest that future studies should also focus more on the improvement of withdrawal symptoms.

Regarding the methodological quality of RCTs, the majority remain at high risk of bias in the blinding of participants and personnel. In terms of random sequence generation, allocation concealment, blinding of outcome assessment, selective reporting, and funding or conflict of interest due to a large amount of under reported information, these entries were ‘unclear’. Future studies can benefit from detailed reporting of trial implementation, pre-registered trial protocols, and truthful reporting of funding.

### Effectiveness of TCM interventions

For point prevalence abstinence, auricular acupressure was more effective than non-drug therapies, but the effectiveness was not significant compared with positive drugs. As for the continuous abstinence rate from 7 days to one year, we find that the longer the follow-up period, the better the effect for continuous abstinence using TCM. Especially in the period of 6 months to one year, TCM intervention has more advantages compared with placebo and non-drug therapies. Uncertainty remains in the studies with more than a 1-year follow-up, so further studies with longer follow-up may be required to substantiate findings. We also find that the TCM intervention combined with varenicline was better than varenicline alone, which suggests that TCM may be used as an adjunctive therapy to conventional treatment. More studies of integrative medicine should be carried out for verification in the future.

TCM intervention is better at alleviating withdrawal symptoms compared with placebo. Meanwhile, the forest plot shows that with the continuous extension of intervention time, the remission effect may be better. It suggests that TCM intervention may be helpful in the long-term smoking cessation process. In the included studies, there is a lack of studies on verifying withdrawal symptoms compared with positive drugs. Future studies are required to obtain a more comprehensive evaluation.

Compared with a placebo group, a TCM intervention had more advantages for reducing relapse rates. But due to the lack of comparisons with other intervention types, more studies are still needed to verify this.

In general, TCM interventions are safe and reliable and can be widely used, but more meticulous and transparent reporting is required in future studies.

### Comparison with previous studies

Previous studies^9^ have not demonstrated that acupoint stimulation is effective for smoking cessation, and systematic reviews published later have replicated this conclusion. Compared with previous studies^34,35^, our review included all TCM smoking cessation interventions and was not limited to a certain therapy. In order to avoid repeated use of data, we did not directly combine or evaluate the included SRs but returned to the included RCTs and pooled the original data.

This study is the first attempt to evaluate SRs and meta-analysis of RCTs using an umbrella review methodology. We also summarized the SRs and RCTs in the field of TCM smoking cessation and found that the current SRs have the problem of duplication, suggesting that reviews should use normative methods for retrieval and avoid research waste. This study is the latest and most comprehensive review of SRs of TCM interventions for smoking cessation. Unless a new TCM modality or large-sample, high-quality RCTs are published, no similar SRs should be conducted in this field.

### Limitations

Regarding quality, most of the included studies did not report basic methodological information, such as allocation concealment and selective reporting, so the quality of the study could not be assessed, and the certainty of the evidence was unclear. Due to the large heterogeneity of interventions and outcomes between studies, data pooling was problematic. Therefore, the small sample size might affect the certainty of the results.

## CONCLUSIONS

Through our review, we found a problem of duplication of topic selection and overlap of included studies in SRs. This demonstrated that research was being wasted and should be avoided in the future. The overall quality of SRs is low, and there is a lack of positive conclusions on efficacy. In terms of clinical characteristics, acupoint stimulation is still the most common intervention. We found TCM had potential advantages in long-term smoking cessation, but further studies are needed to confirm this. More evidence is needed to support the efficacy of TCM on nicotine withdrawal symptoms and relapse rates

## Data Availability

Data sharing is not applicable to this article as no new data were created.
