# Supplementary material for: Traditional Chinese medicine for smoking cessation: An umbrella review of systematic reviews and meta-analysis of randomized controlled trials
Source: Tob Induc Dis. 2023 Nov 15;21:150. doi: 10.18332/tid/174090 (PMC10647068; doi:10.18332/tid/174090)
Supplement: Supplementary file 1 [file TID-21-150-s1.pdf]

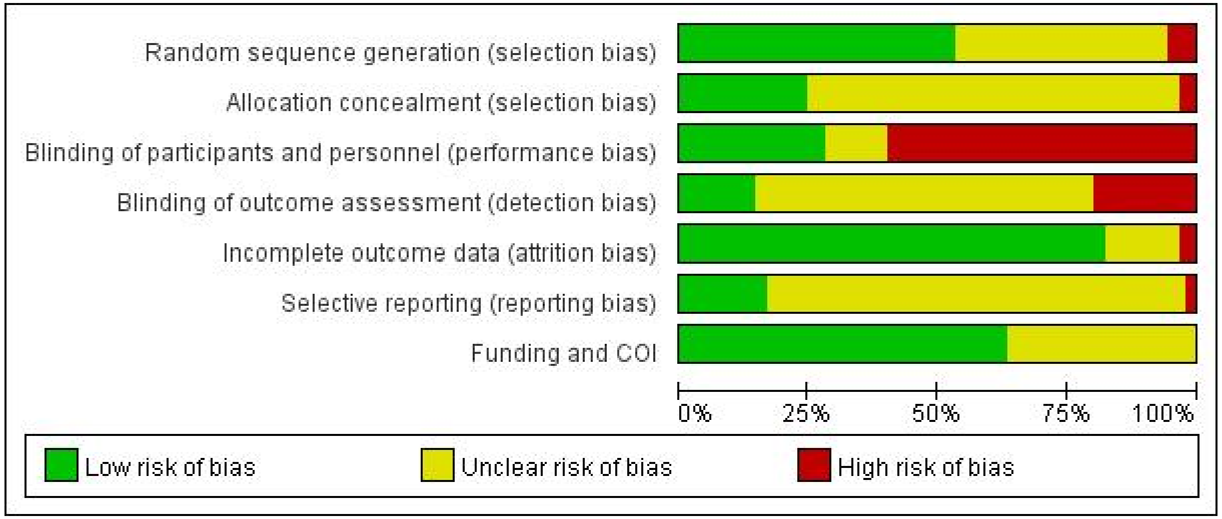

Supplementary Figure 1 Assessment of the risk of bias in RCTs of smoking cessation using TCM

TCM vs Placebo

Study or Subgroup		TCM Mean	TCM SD	TCM Total	Control Mean	Control SD	Control Total	Mean Difference IV, Random, 95% CI
LiuTY 2019	1 W	12.92	4.59	105	13.34	4.1	102	-0.42 [-1.60, 0.76]
LiuTY 2019	2 W	10.12	4.99	105	11.18	4.26	102	-1.06 [-2.32, 0.20]
LiuTY 2019	4 W	5.24	3.2	105	9.7	3.89	102	-4.46 [-5.43, -3.49]

Mean Difference IV, Random, 95% CI

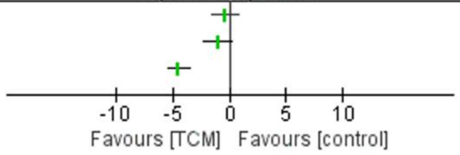

TCM vs NRT

Study or Subgroup		TCM Mean	TCM SD	TCM Total	Control Mean	Control SD	Control Total	Mean Difference IV, Fixed, 95% CI
PengZJ 2015	1 W	3.04	1.61	30	2.86	1.53	30	0.18 [-0.61, 0.97]
PengZJ 2015	2 W	1.92	1.55	30	1.87	1.3	30	0.05 [-0.67, 0.77]
PengZJ 2015	4 W	1.35	1.12	30	1.14	1.08	30	0.21 [-0.35, 0.77]
PengZJ 2015	8 W	1	1.27	30	1.11	1.83	30	-0.11 [-0.91, 0.69]
WuY 2012	1 W	2.57	2.52	30	2.03	1.9	32	0.54 [-0.58, 1.66]
WuY 2012	2 W	2.33	2.55	30	1.5	1.75	32	0.83 [-0.27, 1.93]
WuY 2012	4 W	1.87	2.49	30	1.43	2.05	32	0.44 [-0.70, 1.58]
WuY 2012	8 W	1.5	2.13	30	1.11	1.45	32	0.39 [-0.52, 1.30]
ZhengX 2019	2 W	4.56	0.87	28	4.51	0.79	27	0.05 [-0.39, 0.49]
ZhengX 2019	4 W	3.68	0.79	28	3.7	0.83	27	-0.02 [-0.45, 0.41]
ZhengX 2019	6 W	1.76	0.83	28	1.78	0.8	27	-0.02 [-0.45, 0.41]

Mean Difference IV, Fixed, 95% CI

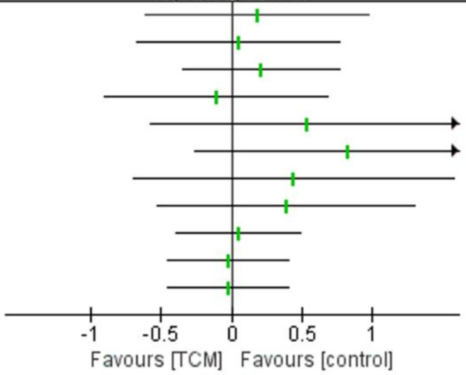

Supplementary Figure 2 Forest plot of RCTs on MNWS in TCM versus placebo and NRT

TCM vs Placebo

Study or Subgroup	TCM Mean	TCM SD	TCM Total	Control Mean	Control SD	Control Total	Mean Difference IV, Fixed, 95% CI
LiuTY 2019 1 W	4.06	1.37	105	4.8	1.15	102	-0.74 [-1.08, -0.40]
LiuTY 2019 2 W	2.75	1.81	105	4.4	1.69	102	-1.65 [-2.13, -1.17]
LiuTY 2019 4 W	1.39	1.68	105	4.09	2.43	102	-2.70 [-3.27, -2.13]

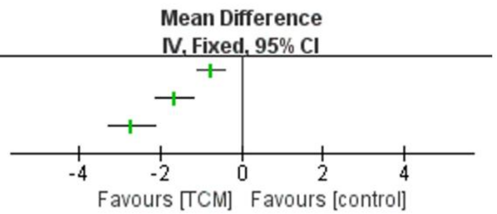

TCM vs NRT

Study or Subgroup	TCM Mean	TCM SD	TCM Total	Control Mean	Control SD	Control Total	Mean Difference IV, Fixed, 95% CI
PengZJ 2015 1 W	3.04	1.61	30	2.86	1.53	30	0.18 [-0.61, 0.97]
PengZJ 2015 2 W	1.92	1.55	30	1.87	1.3	30	0.05 [-0.67, 0.77]
PengZJ 2015 4 W	1.35	1.12	30	1.14	1.08	30	0.21 [-0.35, 0.77]
PengZJ 2015 8 W	1	1.27	30	1.11	1.83	30	-0.11 [-0.91, 0.69]
WuY 2012 1 W	2.57	2.52	30	2.03	1.9	32	0.54 [-0.58, 1.66]
WuY 2012 2 W	2.33	2.55	30	1.5	1.75	32	0.83 [-0.27, 1.93]
WuY 2012 4 W	1.87	2.49	30	1.43	2.05	32	0.44 [-0.70, 1.58]
WuY 2012 8 W	1.5	2.13	30	1.11	1.45	32	0.39 [-0.52, 1.30]
ZhengX 2019 2 W	4.56	0.87	28	4.51	0.79	27	0.05 [-0.39, 0.49]
ZhengX 2019 4 W	3.68	0.79	28	3.7	0.83	27	-0.02 [-0.45, 0.41]
ZhengX 2019 6 W	1.76	0.83	28	1.78	0.8	27	-0.02 [-0.45, 0.41]

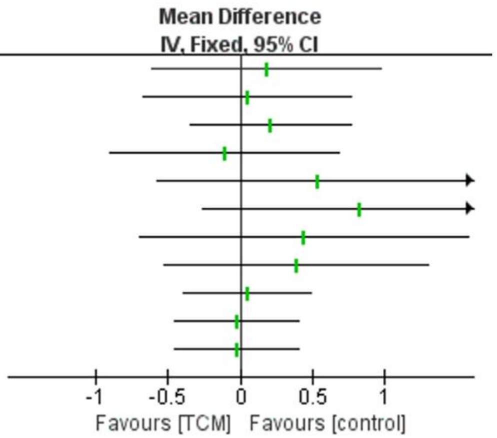

Supplementary Figure 3 Forest plot of RCTs on FTND in TCM versus placebo and NRT

Supplementary Table 1 Searching strategy

English search strategy

#1	smoking cessation
#2	cigarette smoking cessation
#3	tobacco cessation
#4	tobacco smoking cessation
#5	quit smoking
#6	stop smoking
#7	preventing smoking
#8	preventing tobacco
#9	preventing nicotine
#10	nicotine withdrawal
#11	nicotine dependence
#12	tobacco withdrawal syndrome
#13	#1 OR #2 OR #3 OR #4 OR #5 OR #6 OR #7 OR #8 OR #9 OR #10 OR #11 OR #12
#14	Chinese medicine
#15	integrated traditional Chinese and western medicine
#16	Chinese herbs
#17	herb
#18	Chinese patent medicine
#19	decoction
#20	acupuncture
#21	electroacupuncture
#22	auricular acupuncture
#23	laser needle
#24	scalp acupuncture
#25	body acupuncture
#26	nose acupuncture
#27	needle-embedding therapy
#28	Dai Zhen Gao
#29	auricular
#30	auricular point sticking
#31	auricular-plaster

#32	acupressure
#33	plaster on acupuncture points
#34	moxibustion
#35	tuina
#36	massage cupping
#37	scraping
#38	compress
#39	hot compress
#40	smoking given up tea
#41	qi gong
#42	Tai chi
#43	Diet therapy
#44	#14 OR #15 OR #16 OR #17 OR #18 OR #19 OR #20 OR #21 OR #22 OR #23 OR #24 OR #25 OR #26 OR #27 OR #28 OR #29 OR #30 OR #31 OR #32 OR #33 OR #34 OR #35 OR #36 OR #37 OR #38 OR #39 OR #40 OR #41 OR #42 OR #43
#45	randomized controlled trial
#46	controlled clinical trial
#47	randomized
#48	randomly
#49	randomization
#50	RCT
#51	trial
#52	groups
#53	allocat*
#54	blind procedure
#55	crossover procedure
#56	placebo
#57	single blind
#58	double blind
#59	blind
#60	systematic review
#61	#45 OR #46 OR #47 OR #48 OR #49 OR #50 OR #51 OR #52 OR #53 OR #54 OR #55 OR #56 OR #57 OR #58 OR #59 OR #60
#62	#13 AND #44 AND #61

Chinese search strategy

#1	戒烟
#2	烟草
#3	尼古丁
#4	尼古丁戒断
#5	尼古丁依赖
#6	戒烟综合征
#7	#1 OR #2 OR #3 OR #4 OR #5 OR #6
#8	中医
#9	中西医结合
#10	中药
#11	草药
#12	中成药
#13	汤药
#14	针灸
#15	针刺
#16	电针
#17	耳针
#18	激光针
#19	针刀
#20	滚针
#21	头针
#22	体针
#23	腹针
#24	鼻针
#25	腕踝针
#26	埋针
#27	埋线
#28	代针膏
#29	埋豆
#30	耳穴
#31	耳穴贴压
#32	耳压
#33	穴位按压

#34	穴位注射
#35	穴位贴药
#36	针药结合
#37	体穴按摩
#38	艾灸
#39	推拿
#40	按摩
#41	拔罐
#42	刮痧
#43	贴敷
#44	热敷
#45	药物敷贴
#46	戒烟茶
#47	气功
#48	太极
#49	食疗
#50	#8 OR #9 OR #10 OR #11 OR #12 OR #13 OR #14 OR #15 OR #16 OR #17 OR #18 OR #19 OR #20 OR #21 OR #22 OR #23 OR #24 OR #25 OR #26 OR #27 OR #28 OR #29 OR #30 OR #31 OR #32 OR #33 OR #34 OR #35 OR #36 OR #37 OR #38 OR #39 OR #40 OR #41 OR #42 OR #43 OR #44 OR #45 OR #46 OR #47 OR #48 OR #49
#51	随机
#52	系统评价
#53	系统综述
#54	#51 OR #52 OR #53
#55	#7 AND #50 AND #54

Supplementary Table 2 The list of included 88 randomized controlled trials

Study ID	Citation information
Clavel F 1985	[1] Clavel F, Benhamou S, Company-Huertas A, et al. Helping people to stop smoking: randomised comparison of groups being treated with acupuncture and nicotine gum with control group. *Br Med J (Clin Res Ed)*. 1985;291(6508):1538-1539. doi:10.1136/bmj.291.6508.1538
Jiang B 2011	[2] Jiang B, Zuo F, Wu L, et al. Effects of psychological intervention, auricular acupuncture and smoking cessation medication on cigarette smoking behavior in soldiers: a pilot randomized controlled trial. *Chinese Journal of Health Management*. 2011;5(6):353-356. doi:10.3760/cma.j.issn.1674-0815.2011.06.012
Li BL 2016	[3] Li BL. Clinical observation on the effectiveness of Shenmen, Pianli, and Danzhong acupuncture for smoking cessation. *China Academy of Chinese Medical Science*. 2016
Li HY 2019	[4] Li HY, He W. Comparative study of acupoint catgut embedding and bupropion hydrochloride sustained-release tablets for tobacco dependence. *Chinese Acupuncture & Moxibustion*. 2019,39(04):384-388. doi:10.13703/j.0255-2930.2019.04.010.
Li XP 2013	[5] Li XP. Clinical abstinence analysis of 106 severe nicotine dependent smokers. *Journal of Guiyang College of Traditional Chinese Medicine*. 2013,35(03):95-96. doi:10.3969/j.issn.1002-1108.2013.03.0046
Liang WL 2013	[6] Liang WL. Clinical study of acupuncture for smoking withdraw syndrome. Guangzhou University of Chinese Medicine. 2013.
Peng ZJ 2015	[7] Peng ZJ. Clinical efficacy observation and cost-effectiveness ratio analysis of acupuncture and auricular acupoint pressing for abstinence from tobacco addiction. Beijing University of Chinese Medicine. 2015
Wang YY 2017	[8] Wang YY, Liu Z, Wu Y, et al. Efficacy of Acupuncture Is Noninferior to Nicotine Replacement Therapy for Tobacco Cessation: Results of a Prospective, Randomized, Active-Controlled Open-Label Trial. *Chest*. 2018;153(3):680-688. doi:10.1016/j.chest.2017.11.015
Wu Y 2012	[9] Wu Y. Clinical effectiveness of acupuncture and moxibustion on smoking cessation. *China Academy of Chinese Medical Science*. 2015
Zeng XJ 2019	[10] Zeng XJ, Chen ZQ, Wei YF, et al. Efficacy evaluation of acupoint catgut embedding in the treatment of tobacco dependence. *Shenzhen Journal of Integrated Traditional Chinese and Western Medicine*. 2019;29(23):57-59. doi:10.16458/j.cnki.1007-0893.2019.23.027.

Zheng X 2019	[11] Zheng X, Qu NN, Ma LJ, et al. Clinical Study on the Intervention of TCM Quit Smoking Paste Combined with "5A" Program on Tobacco Dependent Population with Syndrome of Phlegm and Blood Stasis. *Journal of Liaoning University of Traditional Chinese Medicine*. 2019;21(3):113-116. doi: 10.13194/j.issn.1673-842x.2019.03.031
Chen JH 2016	[12] Chen JH, Wu ZH, Xu DB. Clinical effectiveness of Qingfei Huatan Liyan Decoction on "smoking lung" and traditional Chinese medicine on smoking cessation. *Nei Mongol Journal of Traditional Chinese Medicine*. 2016;35(13):56. doi:10.3969/j.issn.1006-0979.2016.13.058
Jang SB 2019	[13] Jang S, Lee JA, Jang BH, et al. Clinical Effectiveness of Traditional and Complementary Medicine Interventions in Combination with Nicotine Replacement Therapy on Smoking Cessation: A Randomized Controlled Pilot Trial. *J Altern Complement Med*. 2019;25(5):526-534. doi:10.1089/acm.2019.0009
Li Z 2018	[14] Li Z, Lu XQ, Lin XP. Effect of Combined Therapy of Traditional Chinese and Western Medicines on Moderate to Severe Nicotine Dependence. *Modern Hospital*. 2018;18(7):1046-1048,1051. doi:10.3969/j.issn.1671-332X.2018.07.036
Sun WX 2016	[15] Sun WX. Influence of traditional Chinese medicine quit smoking patch on relapse rate of "nicotine dependence". *Guangming Journal of Chinese Medicine*. 2016;31(3):456. doi:10.3969/j.issn.1003-8914.2016.03.078
White AR 2007	[16] White AR, Moody RC, Campbell JL. Acupressure for smoking cessation--a pilot study. *BMC Complement Altern Med*. 2007;7:8. Published 2007 Mar 14. doi:10.1186/1472-6882-7-8
Baccetti S 2015	[17] Baccetti S , Monechi M V , Da Frè M, et al. Smoking cessation with counselling and Traditional Chinese Medicine (TCM): a randomized controlled trial. *Acupuncture & Related Therapies*. 2015, 3(4):48-54. doi:10.1016/j.arthe.2016.05.002
Bier ID 2002	[18] Bier ID, Wilson J, Studt P, et al. Auricular acupuncture, education, and smoking cessation: a randomized, sham-controlled trial. *Am J Public Health*. 2002;92(10):1642-1647. doi:10.2105/ajph.92.10.1642
Bilici M 2016	[19] Bilici M, Güven S, Köşker S, et al. Electroacupuncture Therapy in Nicotine Dependence: A Double Blind, Sham-Controlled Study. *Noro Psikiyatr Ars*. 2016;53(1):28-32. doi:10.5152/npa.2015.9887
Cai YM 2000	[20] Yiming C, Changxin Z, Ung WS, et al. Laser acupuncture for adolescent smokers--a randomized double-blind controlled trial. *Am J Chin Med*. 2000;28(3-4):443-449. doi:10.1142/S0192415X00000520
Chae YB 2011	[21] Chae Y, Park HJ, Kang OS, et al. Acupuncture attenuates autonomic responses to smoking-related visual cues. *Complement Ther Med*. 2011;19 Suppl 1:S1-S7. doi:10.1016/j.ctim.2010.09.003

Fritz DJ 2013	[22] Fritz DJ, Carney RM, Steinmeyer B, et al. The efficacy of auriculotherapy for smoking cessation: a randomized, placebo-controlled trial. *J Am Board Fam Med*. 2013;26(1):61-70. doi:10.3122/jabfm.2013.01.120157
Georgiou AJ 1998	[23] Georgiou AJ, Spencer CP, Davies GK, et al. Electrical stimulation therapy in the treatment of cigarette smoking. *J Subst Abuse*. 1998;10(3):265-274. doi:10.1016/s0899-3289(99)00005-x
Gillams J 1984	[24] Gillams J, Lewith GT, Machin D. Acupuncture and group therapy in stopping smoking. *Practitioner*. 1984;228(1389):341-344.
He D 1997	[25] He D, Berg JE, Høstmark AT. Effects of acupuncture on smoking cessation or reduction for motivated smokers. *Prev Med*. 1997;26(2):208-214. doi:10.1006/pmed.1996.0125
He D 2001	[26] He D, Medbø JI, Høstmark AT. Effect of acupuncture on smoking cessation or reduction: an 8-month and 5-year follow-up study. *Prev Med*. 2001;33(5):364-372. doi:10.1006/pmed.2001.0901
Huang Y 2012	[27] Huang Y. Clinical observation of treating nicotine dependence with three needles of smoke combined with auricular sticking. Guangzhou University of Chinese Medicine.2012.
Hyun MK 2010	[28] Hyun MK, Lee MS, Kang K, et al. Body Acupuncture for Nicotine Withdrawal Symptoms: A Randomized Placebo-controlled Trial. *Evid Based Complement Alternat Med*. 2010;7(2):233-238. doi:10.1093/ecam/nem179
Kang HC 2005	[29] Kang HC, Shin KK, Kim KK, et al. The effects of the acupuncture treatment for smoking cessation in high school student smokers. *Yonsei Med J*. 2005;46(2):206-212. doi:10.3349/ymj.2005.46.2.206
Kerr CM 2008	[30] Kerr C M, Lowe P B, Spielholz N I. Low level laser for the stimulation of acupoints for smoking cessation: A double blind, placebo controlled randomised trial and semi structured interviews. *Journal of Chinese Medicine*. 2008(86):46-51.
Lambert C 2011	[31] Lambert C, Berlin I, Lee TL, et al. A standardized transcutaneous electric acupoint stimulation for relieving tobacco urges in dependent smokers. *Evid Based Complement Alternat Med*. 2011;2011:195714. doi:10.1093/ecam/nen074
Lee SN 2016	[32] Lee S, Park H. The Effects of Auricular Acupressure on Smoking Cessation for Male College Students. *West J Nurs Res*. 2017;39(3):374-387. doi:10.1177/0193945916660080
Liu TY 2019	[33] Liu TY. Evaluation of clinical efficacy and safety of Chinese traditional medicine quit smoking babu plaster on tobacco dependence (phlegm and blood stasis syndrome). Liaoning University of Chinese Medicine. 2019.
Ma W 2014	[34] Ma W, Xue JC. Effects of acupuncture and moxibustion on sialic acid and C-reactive protein in patients who quit smoking. *Modern Journal of Integrated Traditional Chinese and Western Medicine*. 2014;(30):3337-3338.doi:10.3969/j.issn.1008-8849.2014.30.011.

Sood A 2010	[35] Sood A, Ebbert JO, Prasad K, et al. A randomized clinical trial of St. *John's wort for smoking cessation. J Altern Complement Me d*. 2010;16(7):761-767. doi:10.1089/acm.2009.0445
Steiner RP 1982	[36] Steiner RP, Hay DL, Davis AW. Acupuncture therapy for the treatment of tobacco smoking addiction. *Am J Chin Med*. 1982;10(1-4):107-121. doi:10.1142/S0192415X82000178
Sun X 2009	[37] Sun X. Clinical study on 24 cases of smoking dependence cured by Traditional Chinese medicine kangyangjian. 2009 National Sym posium on Drug Abuse Prevention. 2009
Waite NR 1998	[38] Waite NR, Clough JB. A single-blind, placebo-controlled trial of a simple acupuncture treatment in the cessation of smoking. *Br J Gen Pract*. 1998;48(433):1487-1490.
White AR 1998	[39] White AR, Resch KL, Ernst E. Randomized trial of acupuncture for nicotine withdrawal symptoms. *Arch Intern Med*. 1998;158(20): 2251-2255. doi:10.1001/archinte.158.20.2251
LI YK 2010	[40] Wing Y K, Lee A, Wong E L Y, et al. Auricular Acupressure for Smoking Cessation: A Pilot Randomized Controlled Trial. *Medi cal Acupuncture*. 2010, 22(4):265-271. doi: 10.1089/acu.2010.0763
Wu TP 2007	[41] Wu TP, Chen FP, Liu JY, Lin MH, Hwang SJ. A randomized controlled clinical trial of auricular acupuncture in smoking cessatio n. *J Chin Med Assoc*. 2007;70(8):331-338. doi:10.1016/S1726-4901(08)70014-5
Yeh ML 2009	[42] Yeh ML, Chang CY, Chu NF, Chen HH. A six-week acupoint stimulation intervention for quitting smoking. *Am J Chin Med*. 200 9;37(5):829-836. doi:10.1142/S0192415X09007314
Zhang AL 2013	[43] Zhang AL, Di YM, Worsnop C, et al. Ear acupressure for smoking cessation: a randomised controlled trial. *Evid Based Complemen t Alternat Med*. 2013;2013:637073. doi:10.1155/2013/637073
Zhao HJ 2018	[44] Zhao HJ, Ren XL, Chang XH, et al. Effect of self-made Chinese herb application by plastering on acupoint for smoking cessation. *Journal of Gansu University of Chinese Medicine*. 2018;35(1):77-80. doi: 10.16841/j.issn1003-8450.2018.01.21
Bai L 2001	[45] Bai L, Ren BQ. Effect of needling Ren's smoking cessation zone on cotinine content. *Mordern Rehabilitation*. 2001;5(3):144. doi: 1 0.3321/j.issn:1673-8225.2001.03.120
Chen H 2019	[46] Chen H. The influence of smoking on lung function and the effect of qingfei tea on smoking cessation. Hebei North University. 2 019.
Gilbey V 1977	[47] Gilbey V, Neumann B. Auricular acupuncture for smoking withdrawal. *American Journal of Acupuncture*. 1977, 5(3):239-247.

Gong ZY 2008	[48] Gong ZY, Tang SJ, Liu XL, et al. The influence of smoking on lung function and the effect of qingfei tea on smoking cessation. Hebei North University. 2019.
Li Y 2009	[49] Li Y, Shen TL, Cao LY, et al. Experimental Research of Radom, Comparison and Single Blind on Quitting Smoking by Auricular-plaster Therapy. *Jilin Journal of Traditional Chinese Medicine*. 2009;29(6):505-506. doi: 10.3969/j.issn.1003-5699.2009.06.027
Reif MH 1999	[50] Hernandez-Reif M, Field T, Hart S. Smoking cravings are reduced by self-massage. *Prev Med*. 1999;28(1):28-32. doi:10.1006/pmed.1998.0372
Silva RP 2014	[51] Silva Rde P, Chaves Ede C, Pillon SC, et al. Contributions of auriculotherapy in smoking cessation: a pilot study. *Rev Esc Enferm USP*. 2014;48(5):883-890. doi:10.1590/s0080-623420140000500015
Wang YX 2006	[52] Wang YX. Clinical study on continuous stimulation of auricular points to quit smoking. *China Journal of Traditional Chinese Medicine and Pharmmacy*. 2006;004(5).
Zhang Y 2017	[53] Zhang Y. Clinical observation on 30 cases of smoking cessation syndrome treated by acupuncture (combined with snap acupuncture). *Heilongjiang Journal of Traditional Chinese Medicine*. 2017;46(2):60.
Chu C 2013	[54] Chu C. Study on the application of acupoint reinforcing and reducing in the clinical treatment of smoking cessation. Nanjing University of Chinese Medicine. 2013.
Guo B 2020	[55] Guo B, Gong LL. Effect Analysis of Traditional Chinese Medicine Nursing of Auricular Acupoint Pressing Combined with"5A"Method on Smoking Cessation in Patients with Angina Pectoris. *Guide of China Medicine*. 2020;18(22):153-154.
He HJ 2019	[56] He HJ. Clinical study on smoking cessation with fire needle. Guangzhou University of Chinese Medicine. 2019.
Lee EJ 2019	[57] Lee EJ. Auricular Acupressure and Positive Group Psychotherapy With Motivational Interviewing for Smoking Cessation. *Holist Nurs Pract*. 2019;33(4):214-221. doi:10.1097/HNP.0000000000000333
Lee EJ 2020	[58] Lee EJ. The Effect of Auricular Acupressure and Positive Group Psychotherapy With Motivational Interviewing for Smoking Cessation in Korean Adults. *Holist Nurs Pract*. 2020;34(2):113-120. doi:10.1097/HNP.0000000000000348
Leung JP 1991	[59] Tobacyk, J, Miller, M. Comment on “Maslow’s Study of Self-Actualization.” *Journal of Humanistic Psychology*. 1991;31(4), 96–98. doi:10.1177/0022167891314007
Li LB 2011	[60] Li LB, Sun JW, Xu DL, et al. The Approach of Ear Acupuncture Combining to Psychological Intervention with the Health Belief Mode in Community Tobacco Control. *Zhejiang Journal of Preventive Medicine*. 2011;23(9):23-25. doi: 10.3969/j.issn.1007-0931.2011.09.

	007
Sun CX 2000	[61] Sun CX. Roles of Psychological Nursing Played in the Course of Auricle Point Applying to Help Individuals Giving up Smoking. *Shanxi Nursing Journal*. 2000;14(2):69-70. doi: 10.3969/j.issn.1009-6493.2000.02.016
Wan ZX 2021	[62] Wan ZX, Yao YL, Chen JL, et al. Clinical effect of auricular point pressing seeds combined with cognitive behavioral therapy on reducing relapse rate of smoking cessation. *Clinical Research and Practice*. 2021;6(10):120-122. doi:10.19347/j.cnki.2096-1413.202110038
Yavagal PC 2021	[63] Yavagal PC, L N. Efficacy of Laser Auricular Acupuncture for Smoking Cessation: A randomised controlled trial. *Sultan Qaboos U niv Med J*. 2021;21(2):e275-e281. doi:10.18295/squmj.2021.21.02.017
Yeh ML2014	[64] Yeh ML, Wang PL, Lin JG, Chung ML. The effects and measures of auricular acupressure and interactive multimedia for smoking cessation in college students. *Evid Based Complement Alternat Med*. 2014;2014:898431. doi:10.1155/2014/898431
Zhao L 2017	[65] Zhao L. Clinical observation of auricular acupoint pressing combined with cognitive behavioral therapy (CBT) in the treatment of n icotine dependence. Shanxi University of Chinese Medicine. 2017
Chai X 2019	[66] Chai X, Yang JS, Liu Z, et al. Effect of the different smoking cessation regimens with acupuncture on smoking withdrawal and th eir influence factors: a multi-center randomized controlled trial. *Chinese Acupuncture & Moxibustion*. 2019;39(12):1255-1261. doi: 10.137 03/j.0255-2930.2019.12.001
Chapelon FC 1997	[67] Clavel-Chapelon F, Paoletti C, Benhamou S. Smoking cessation rates 4 years after treatment by nicotine gum and acupuncture. *Prev Med*. 1997;26(1):25-28. doi:10.1006/pmed.1996.9997
Cottraux JA 1983	[68] Cottraux JA, Harf R, Boissel JP, et al. Smoking cessation with behaviour therapy of acupuncture--a controlled study. *Behav Res Th er*. 1983;21(4):417-424. doi:10.1016/0005-7967(83)90011-6
Gu ZR 2007	[69] Gu ZR. Clinical summary and discussion of 400 cases of quitting smoking with acupoint therapy. The 13th National Symposium o n Smoking and Health and the third FCTC Forum. 2007
Parker LN 1977	[70] Gilbey V, Neumann B. Auricular acupuncture for smoking withdrawal. *American Journal of Acupuncture*. 1977;5(3):239-247.
Qin YB 2020	[71] Qin YB, Qu NN, Zheng X, et al. Clinical observation of Chinese herbal quit smoking Babu Patch combining acupuncture treating t obacco dependence with phlegm and blood stasis syndrome. *China Journal of Traditional Chinese Medicine and Pharmacy*. 2020;35(11): 5868-5872.

Han Y 2006	[72] Han Y. Treatment of 42 cases of smoking cessation syndrome by acupuncture combined with auricular acupoint pressing. *Journal of Clinical acupuncture and moxibustion*. 2006; 22(11):16-16. doi: 10.3969/j.issn.1005-0779.2006.11.011
Liu D 2015	[73] Liu D, Shao YR, Liu F. Clinical Study on Acupuncture plus Auricular Point Sticking in Improving Tobacco Withdrawal Symptoms After Smoke Cessation. *Shanghai Journal of Acupuncture and Moxibustion*. 2015;(7):629-631. doi: 10.13460/j.issn.1005-0957.2015.07.0629
Wang DF 2021	[74] Wang DF, Wang LM, Yu J, et al. Research on the Clinical Effect of Auricular Point Sticking on 160 Cases of Smoking Cessation Based on the Method of Auricular Point Sticking with Cow Herb Seed. *Chinese Primary Health Care*. 2021;35(6):76-78. doi: 10.3969/j.issn.1001-568X.2021.06.0023
Wang WH 2013	[75] Wang WH, Song YX. Clinical Trial of Acupuncture plus Moxibustion for Smoke Cessation. *Shanghai Journal of Acupuncture and Moxibustion*. 2013;32(4):287-288. doi: 10.3969/j.issn.1005-0957.2013.04.287
Wang XJ 2013	[76] Wang XJ, Xu B, Li JW, et al. Observation on the effectiveness of sticking tea on smoking cessation. *Shanxi Journal of Traditional Chinese Medicine*. 2013;34(4):430-433. doi: 10.3969/j.issn.1000-7369.2013.04.027
Zhou JH 2010	[77] Zhou JH, Wang RF. Observation on Effect of Electrical Acupuncture and Ear Pressure with Magnetic Bead on Smoking Abstention. *Journal of Nanjing University of Traditional Chinese Medicine*. 2010;26(1):79-80. doi: 10.3969/j.issn.1000-5005.2010.01.027
Zhang JM 1989	[78] Zhang JM, Tan SG, Yu JB. 120 cases of smoking cessation with auricular acupoint and body acupoint pressure. *Modern Traditional Chinese Medicine*. 1989;(4):28-29.
Zhang QG 2004	[79] Zhang QG, Gu KB, Wang D, et al. Treatment of tobacco dependence with wrist-ankle acupuncture:A report of 30 cases. *Journal of Integrative Medicine*. 2004;2(6):444-444,480.
Zhang X 2016	[80] Zhang X. Clinical observation on the treatment of Tobacco dependence with Liver Stagnation and Qi Stagnation by acupoint acupuncture of Liver Meridian and mother. Heilongjiang University of Chinese Medicine. 2016
Cai ZB 2022	[81] Cai ZB, Zeng JC, Luo ZK, et al. Clinical randomized controlled trial of acupuncture combined with auricular acupoint compression in the treatment of tobacco dependence. *Chinese Journal of Traditional Chinese Medicine.* 2022;37(11):6864-6867.
Chen SM 2022	[82] Chen SM, Liu ZY, Ji J, et al. Auricular acupoint pressure combined with percutaneous acupoint electrical stimulation for smoking cessation: a randomized controlled trial. *Chinese Journal of Acupuncture*. 2022;42(11):1235-1239. doi:10.13703/ J.0255-2930.20220104-k0001.

Chandrabhaga SV 2021	[83] Velangi CS, Yavagal PC, Nagesh L. Role of auricular laser acupuncture and psychological counseling in reducing nicotine dependen ce due to smoking: A randomized controlled trial. *Indian J Public Health*. 2021;65(3):243-249. doi:10.4103/ijph.IJPH_810_20
Dai RJ 2022	[84] Dai R J. A randomized controlled trial of acupuncture for smoking cessation based on Bayesian mesh meta-analysis. Gansu universi ty of traditional Chinese medicine, 2022.
Ji J 2022	[85] Ji J. Smoking cessation effect of acupuncture with different frequency based on nicotine metabolic rate. China Academy of Traditio nal Chinese Medical Sciences, 2022.
Kuang HJ 2021	[86] Kuang HJ, Fu SS, Huang Q, et al. Effects of auricular point sticking combined with transcranial direct current stimulation on tobac co dependence. *Journal of Rehabilitation.* 2021;31(05):381-388.
Zhang N 2022	[87] Zhang N, She YL, Lin GH. Columns of acupuncture through thorn li 5 applied to study the clinical effect of nicotine addiction pa tients. *Big doctor.* 2022;7(17):79-81.
Zeng XJ 2021	[88] Zeng X J. Effects of acupoint catgut embedding combined with cognitive behavioral therapy on smoking cessation rate, lung functio n and quality of life in tobacco dependent patients. *The Chinese and foreign medical research.* 2021;(24):169-171.

Supplementary Table 3 Characteristics of the 2 protocols of TCM for smoking cessation

**Study ID**	**Country / region**	**Settings**	**Target sample size**	**Participants**	**Age/year**	**Smoking year**	**Daily cigarette consumption**	**Comparison (Duration of intervention)**	**Outcomes**	**Follow-up**	**Predicted completion time**	**Publish or not**
NCT02298127	Hong Kong, China	School of Nursing, The Hong Kong Polytechnic University	90	Be able to communicate in Cantonese；Currently reside in Hong Kong and expect to continue to do so for the next 6 months；Have access to a telephone	≥18	Currently smoking at least one cigarette per day in the past 30 days		Auricular acupressure vs Sham auricular acupressure (4 w)	7-day point prevalence by self-report; 7-day point prevalence by self-report, validated by biochemical measure; Withdrawal symptoms	NR	June 2016	Not found
NCT01389622	Canada/Ontario	Queen's Family Health Team	60	Participants with confirmed habit of tobacco smoking	18-75	≥6 m	NR	National Acupuncture Detoxification Association (NADA) points + Random points + digital pressure with urgedigital pressure vs Advice + support (6 w)	Spot 7-day cessation rate; Cessation Rate (CAR); ·End-expiratory carbon monoxide levels (CO); Adverse effects; Stress level according to the Stress Check List (SCL); ·Nicotine withdrawal symptoms according to Minnesota Nicotine Withdrawal Scale (MNWS)	13 w	January 2016	Not found

Noted: NR=Not reported; w=week(s); m=month(s)

Supplementary Table 4 Summary of characteristics of 13 included systematic reviews of TCM for smoking cessation

Study ID	TCM group intervention	Control group intervention	Primary outcomes	Secondary outcomes	Conclusion
Ashenden R 1997[22]	Acupuncture, acupuncture + electro acupuncture, individual hypnosis, group hypnosis, self-monitoring, group behavior therapy, ankle acupuncture, acupuncture + nicotine gum	Sham acupuncture + electro acupuncture, nicotine gum, locked cigarette case, individual hypnosis, group hypnosis, sham acupuncture, self-monitoring, group behavior therapy, sham acupuncture + placebo gum	Smoking cessation rates	NR	Potential effective
Cheng HM 2012[23]	Acupuncture, auricular acupuncture, auricular acupressure, electroacupuncture, electrode placement, placebo acupuncture, placebo auricular acupuncture, placebo electroacupuncture, placebo medicine, waiting list, behavior therapy, group therapy, education, nicotine gum<br>Acupoints: Shenmen, sympathetic, lung, kidney, liver, hegu, shuaigu, qiuhou, bitong, shuaigou,gall bladder, lung, shuaigu, tongziliao, timme, mouth, lung, subcortex, endocrine, heart	Placebo acupuncture, placebo auricular acupuncture, placebo electroacupuncture, placebo medicine, waiting list, behavior therapy, group therapy，nicotine gum, placebo nicotine gum, lactose capsules, relaxation, self-monitoring	Smoking cessation rate and daily cigarette consumption	NR	Effective

Study ID	TCM group intervention	Control group intervention	Primary outcomes	Secondary outcomes	Conclusion
Di YM 2014[24]	Combined ear and body points, methods of stimulation included needles, pressure, electricity, laser, and combinations of these	Sham acupuncture/acupressure, wait-list or no intervention, oral placebo, medical therapy	Cigarette smoking cessation	NR	Potential effective
Wang JH 2019[25]	Transdermal acupuncture by needles with or without additional application of electrostimulation or laser therapy. The acupuncture points used could be on the body, ear, face or head, delivered as a monotherapy or in combination with other interventions (NRT, acupressure, etc.).	No intervention / waiting list, placebo,	Short-, mid-, and long-term abstinence from smoking	Nicotine withdrawal symptoms (NWS), the fagerstrom test for nicotine dependence (FTND), the beck depression inventory (BDI) and the beck anxiety inventory (bai), exhaled carbon monoxide (CO) level, cotinine content (in serum, urine or saliva), daily cigarette consumption, craving for cigarette, heaviness of smoking index (HSI), brief questionnaire of smoking urges (QSU-brief), and adverse events if reported	Potential effective

Study ID	TCM group intervention	Control group intervention	Primary outcomes	Secondary outcomes	Conclusion
Kim SS 2012[26]	Counseling, TCM (ear point seed pressure versus acupuncture), pharmacotherapy (nicotine sublingual tablet), a combination of pharmacotherapy and counseling, physician advice	Nicotine replacement therapy, placebo	Abstinence rate	NR	Potential effective
Dai RJ 2021[27]	Auricular acupressure; acupuncture therapy; acupuncture combined with auricular acupressure	Sham acupuncture therapy; nicotine replacement therapy; sham auricular acupressure; auricular acupressure; acupuncture combined with auricular acupressure	Short-term and long-term abstinence rates. breath carbon monoxide, urinary cotinine, or both.	The change of the scores of fagerstrom test for nicotine dependence (FTND) and daily smoking prior to and after treatment	Potential effective
Tahiri M 2012[28]	Acupuncture	NR	The most rigorous criterion uses the most conservative outcome reported in any given randomized controlled trial.	NR	Potential effective

Study ID	TCM group intervention	Control group intervention	Primary outcomes	Secondary outcomes	Conclusion
White AR 2014[9]	Facial acupuncture; auricular acupuncture alone; continuous stimulation, either needle or pressure device; combined body and auricular acupuncture; continuous stimulation with either indwelling needles; facial, body, indwelling, and sham auricular acupuncture in different groups; acupressure alone; electrostimulation given over the mastoid bone; electrostimulation to the ear; continuous acupressure stimulation	Points for the control group (neurological approach); auricular point 'kidney'; vitamins and herbal medicine, extract of hawthorn; locked cigarette case controlled by a time-switch; placebo capsules; 'medical treatment' consisting of advice, a benzodiazepine drug, lobeline, and a 'detoxicant'; counselling; psychological approach; waiting list; placebo capsules; group therapy; no-treatment control arm; behavioral therapy	Complete abstinence from smoking. The review has not been limited to studies where the outcome was confirmed biochemically.	NR	Potential effective
White A 2006[29]	3 respiratory, aggression, sensory + psychotherapy; 2 bilateral lung; lung; 6 seeds, body electroacupuncture, ear acupuncture; 4 bilateral lung + shenmen; 1 suture + bead, earlobe; 2 sutures + beads, lung and shenmen; 2 lung, hunger, same with electroacupuncture one week later, cut-off stud; 2 lung, shenmen; 2 lung, stomach, body acupuncture; 4 seeds mouth, lung, subcortex, shenmen; zero, lung; 1 seed lung, counselling, electroacupuncture	Psychotherapy + illustrations; psychotherapy + medication; 2 studs, ‘incorrect’ point (kidney) bilateral; 1 stud, ‘incorrect’ pt; group therapy; 6 seeds in ‘incorrect’ points plus 6 sessions of ear acupuncture and body electroacupuncture, to ‘incorrect’ points; behavioral therapy; waiting list; suture ‘incorrect’ point; hypnosis, individual; hypnosis, group; no treatment; electroacupuncture; body acupuncture to ‘incorrect’ points (no press needles), and one session of ear acupuncture; advice; body	From the earliest time point available after treatment as this would be likely to show the greatest effect of treatment (and the least influence of relapse).	NR	Potential effective

Study ID	TCM group intervention	Control group intervention	Primary outcomes	Secondary outcomes	Conclusion
		acupuncture; nasal acupuncture; seeds to 'incorrect' points on knee (mean duration 7 days)			
Liu Z 2015[30]	Acupuncture, auricular acupressure + body acupuncture, auricular electrostimulation, auricular acupressure combined with psychological intervention, laser, waist and ankle acupuncture, auricular acupressure, electroacupuncture combined with auricular acupressure, acupuncture + moxibustion	Placebo drug, NRT, sham acupuncture, psychological intervention, sham laser, body acupuncture, auricular acupressure, placebo acupuncture	NR	NR	Potential effective
White AR 1999[31]	Facial; auricula; face; electroacupuncture; body	Sham; nicotine/placebo gum; behavioral; body, for relaxation; waiting list; nicotine gum; time cigarette case; placebo	complete cessation of smoking at three time points	NR	Potential effective

Study ID	TCM group intervention	Control group intervention	Primary outcomes	Secondary outcomes	Conclusion
Liu ZY 2023[32]	Body acupuncture, auricular acupressure, acupoint application, laser acupuncture	Nicotine patches, sham acupuncture, cognitive behavioral therapy	MNWS, FTND, HIS, QSU, HAMD, withdrawal rate, effective rate	NR	Potential effective
Kuang HJ 2022[33]	Acupuncture, acupuncture + auricular acupressure, auricular acupressure + counselling, fire needling, ear acupuncture, ear acupuncture + counselling, acupuncture + TCM patch,	Nicotine patch, sham acupuncture, education, sham ear acupuncture, sham ear acupuncture + education, sham auricular acupressure + education	Continuous abstinence rate, point abstinence rate (abstinence rate based on blood and urine cotinine concentration as evaluation criteria was preferred, followed by self-reported abstinence rate of quitters)	Effective rate, FTND score, MNWS score, safety evaluation.	Potential effective

Noted: NR=Not reported; TCM=Traditional Chinese medicine; NRT=Nicotine replacement therapy; FTND=Fagerstrom Test of Nicotine Dependence; MNWS=Minnesota Nicotine Withdrawal Symptoms Scale; QSU-Brief= Brief Questionnaire of Smoking Urges; HAMD=Hamilton

Depression Scale; HIS=heaviness of smoking index

Supplementary Table 5 Characteristics of RCTs of traditional Chinese medicine smoking cessation

Study ID	Sample size (Total/in group: M/F)	Participants	TCM interventions	Comparison (Duration of intervention)	Outcomes	Follow-up
TCM vs. Bupropion, 1 trial						
Li HY 2019	100<br>I:41/8<br>C:40/8	Adult smokers	Acupoint catgut embedding therapy	Acupoint catgut embedding therapy *vs*. Bupropion hydrochloride sustained-release tablets (7 w)	Abstinence rate, FTND, Medical CO detection; treatment compliance; adverse events and adverse event incidence	None
TCM vs. Nicotine replacement therapy (NRT), 12 trials						
Chen SM 2022	200<br>I:99/1<br>C:98/2	Adult smokers	Transcutaneous electrical acupoint stimulation + Auricular acupressure	Transcutaneous electrical acupoint stimulation + Auricular acupressure vs Nicotine patch (8w)	Abstinence rate, FTND, MNWS, adverse event, compliance	16 w
Clavel F 1985	651<br>I1:224<br>I2:205<br>C:222	Adult smokers	Acupuncture	Acupuncture *vs*. Nicotine gum *vs*. Minimal intervention (consisting of a cigarette case with a lock controlled by a time switch, which they could regulate at will) (1m)	Abstinence rate	13 m
Li BL 2016	50<br>I:25<br>C:25	Adult smokers	Acupuncture	Acupuncture *vs*. Nicotine patch (8 w)	Abstinence rate, FTND, HSI, cobalt content, urinary cotinine content, adverse event	6 m

Li XP 2013	106<br>I1:30/6<br>I2:33/7<br>C:21/9	Adult smokers	TCM tablet	TCM tablets *vs*. NRT *vs*. NRT + psychological intervention (50 d)	Abstinence rate, relapse rate	5, 10, 15 w
Liang WL 2013	60<br>I:30<br>C:30	Adult smokers	Acupuncture	Acupuncture *vs*. Nicotine patch (2 0 d)	Smoking cessation symptom scale, total effective rate	NR
Peng ZJ 2015	60<br>I:27/3<br>C:26/4	Adult smokers	Acupuncture and auricular acupoints	Acupuncture + auricular acupunctu re *vs*. NRT (8 w)	FTND, HSI, abstinence rate, health economic indicators	NR
Wang YX 2006	204	Adult smokers	Auricular acupressure	Auricular acupressure *vs*. Oral sm oking cessation candy (20 d)	Effectiveness rate	NR
Wang YY 2017	300<br>I1:100<br>I2:100<br>C:100	Adult smokers	Acupuncture, auricular acupressure	Acupuncture + auricular acupressu re *vs*. NRT (8 w)	24-week continuous abstinence; 24-hour point prevalence abstinence	12 w
Wu Y 2012	90<br>I:27/3<br>C:30/2	Adult smokers	Auricular acupressure + body acupuncture	Auricular acupressure + body acu puncture *vs*. Nicotine patch (8 w)	Abstinence rate, FTND, HSI, MNWS, smoking craving questionnaire scale (QSU-Brief), smoking reduction	20 w

Zhang Y 2017	60<br>I:30<br>C:30	Patient willing to quit smoking	Acupuncture	Acupuncture + intradermal needlin g vs Nicotine gum (30d)	Clinical effectiveness	NR
Zhang N 2022	82<br>I:36/5<br>C:37/4	Adult smokers	Acupuncture + Auricular acupressure	Acupuncture + Auricular acupress ure vs Nicotine patch (8w)	Expiratory CO, daily cigarette consumption, Abstinence rate, HAMA, adverse event	NR
Zheng X 2019	60<br>I:29/1<br>C:28/2	Adult smokers	TCM quit smoking patch + 5A	TCM quit smoking patch acupress ure +5a *vs.* Nicotine patch +5a (6 w)	Abstinence rate, relapse rate, FTND, MNWS, expiratory CO, daily cigarette consumption	2 w
TCM + NRT vs. NRT, 2 trials						
Jang SB 2019	41<br>I:20/0<br>C:20/1	Adult smoker	TCM therapy: body (auricular) acupuncture + aromatherapy	TCM + NRT + consulting vs. NR T + consulting (4 w)	4-week continuous abstinence, 7-day prevalence abstinence; number of cigarettes per day; tobacco craving; FTND; MNWS; expiratory CO content; breathing capacity	6 m
White AR 2007	23<br>I1:4/2<br>I2:1/5<br>C:0/7	Adult smokers	Auricular acupressure	Binaural acupuncture beads + NR T *vs*. Monaural acupuncture beads + NRT *vs*. NRT (4 w)	NRT consumption; nicotine relapse symptoms; MPSS; 4-week abstinence rate; adverse event	2 w

TCM + Varenicline vs. Varenicline, 1 trial						
Sun WX 2016	60<br>I:26/4<br>C:25/5	Adult smokers	Quit smoking patch applied to "quit smoking acupoints"	Smoking cessation patch + oral varenicline + doctor's encouragement *vs.* Oral varenicline + doctor's encouragement (2 w)	Relapse rate	3 m
TCM vs. Placebo, 33 trials						
Baccetti S 2015	477<br>I1:57/102<br>I2:97/103<br>C:56/100	Adult smokers	Medicated acupuncture, plum-blossom needle or auricular acupuncture	Sham TCM + psychological support *vs.* Real TCM + psychological support *vs.* Real TCM (nr)	Proportion of participants additional stopped smoking, and FTND	End of treatment, 6 m, 1 y
Bier ID 2002	141<br>I1：45<br>I2：38<br>C:58	Adult smokers	Acupuncture	Acupuncture *vs.* Acupuncture + 5 weeks of cessation education *vs.* Sham acupuncture + 5 weeks of cessation education (4 or 5 w)	Change in cigarette consumption; abstinence rate	3, 6, 12, 15 and 18 m
Bilici M 2016	164<br>I:65/19<br>C:55/25	Adult smokers	Electroacupuncture	Electroacupuncture *vs.* Sham electroacupuncture (4 w)	FNDT change in 4, 8 weeks ; daily cigarette consumption change in 4, 8 weeks ; CO content change in 4, 8 weeks	4 w
Cai YM 2000	268<br>I:128<br>C:140	Teenage smoker	Laser Needle	Laser needle *vs.* Placebo (4 w)	Abstinence rate, daily cigarette consumption	3 m

Chae YB 2011	29<br>I:15/0<br>C:14/0	Adult smokers	Acupuncture	Acupuncture *vs*. Sham acupuncture (3 d)	Expiratory CO content, heart rate variability, response to smoking-related visual cues	NR
Dai RJ 2022	88<br>I1:21/1<br>I2:22/0<br>C1:21/1<br>C2:22/0	Adult smokers	Acupuncture	Acupuncture + Nicotine Patch vs Acupuncture + Sham Nicotine Patch vs Sham Acupuncture + Nicotine Patch vs Sham Acupuncture + Sham Nicotine Patch (8 weeks)	Abstinence rate, relapse rate, exhaled CO, FTND, MNWS, QSU-Brief	16 w
Fritz DJ 2013	125<br>I:16/48<br>C:14/47	Veteran	Auricular electrostimulation	Auricular needle electrostimulation *vs*. Sham auricular needle electrostimulation (5 w)	Point prevalence abstinence in 3, 6 weeks , nicotine withdrawal symptoms, perceived stress, self-reported smoking frequency	3, 6 w
Georgiou AJ 1998	265<br>I1:24<br>I2:30<br>I3:24<br>I4:30<br>C1:24<br>C2:30<br>C3:24<br>C4:30	Adult smokers	Electrode acupoint stimulation	Electrical stimulation *vs*. Comfort electrical stimulation (7 d)	Daily cigarette consumption, abstinence rate, expiratory CO content	12 w

Gillams J 1984	55<br>I:28<br>C:27	Adult smoker who smoked >50 cigs/week for 5 year	Auricular acupoints intradermal needling	Auricular acupoints intradermal needling vs non-specific acupoints stimulation (4w)	NA	NA
Gilbey V 1997	92<br>I:44<br>C:48	Smoker who smoked >15 cigs/day for 3 years	Auricular acupoints intradermal needling	Auricular acupoints intradermal needling vs inactive auricular point ('Kidney') stimulation（1w）	NA	NA
He D 1997	46<br>I:8/18<br>C:10/10	Adult smokers	Body electroacupuncture, auricular acupuncture, auricular acupressure	Acupuncture *vs*. Sham acupuncture (3 w)	Daily cigarette consumption, smoking-related blood indicators, tobacco taste, and tobacco craving	NR
He D 2001	46<br>I:8/18<br>C:10/10	Adult smokers	Body electroacupuncture, auricular acupuncture, auricular acupressure, body electroacupuncture, auricular acupuncture, auricular acupressure	Specialized smoking-cessation acupoint *vs*. Ineffective smoking cessation acupoint (3 w)	Daily cigarette consumption; physiological and biochemical indices	End of treatment, 8 m, 5 y

Huang Y 2012	60<br>I:27/3<br>C:25/5	Adult smokers	Smoke three needles combined with auricular acupressure	Smoke three needles combined wi th auricular acupressure *vs*. Smoke three needles combined with auri cular acupressure + placebo acupu ncture	Abstinence rate, cigarette smoking, FTND, and tobacco dependence self-rating scale	3 m
Hyun MK 2010	80<br>I:36/2<br>C:39/3	Adult smokers	Acupuncture	Acupuncture *vs*. Sham acupuncture (2 w)	NWS, BDI; BAI	2 w
Kang HC 2005	238<br>I:159/0<br>C:79/0	High school smoker	Auricular acupuncture	Auricular acupuncture *vs*. Sham ac upuncture (4 w)	Abstinence rate; tobacco craving; daily cigarette consumption	NR
Kerr CM 2008	340	Smoker	Laser stimulation of acupoints	Laser stimulation + sham treatmen ts (inactive probe) vs Laser stimul ation vs sham treatments (day 1, 3, 7, 14)	Relapse rate, physical symptoms	3, 6, 8m
Lambert C 2011	98<br>I1:20/0<br>I2:18/2<br>I3:13/7<br>I4:15/6<br>C:15/2	Adult smokers	Electroacupuncture	Electroacupuncture of different int ensities *vs*. Sham electroacupunctu re (26 h)	Tobacco craving; blood pressure and other physiological and biochemical indices; expiratory CO content	NR

Lee SN 2016	53<br>I:27/0<br>C:26/0	Male college smoker	Ear pressure beads	Auricular acupressure beads *vs*. Placebo group (6 w)	Expiratory CO content; FTND	6 w
Lee EJ 2019	55<br>I1:17/1<br>I2:16/3<br>C:18/0	Medical school smoker	Auricular acupressure	Auricular acupressure + positive group psychotherapy motivational interview *vs*. Placebo auricular acupressure + positive group psychotherapy motivational interview *vs*. Self-help smoking cessation (6 w)	1-year abstinence rate, tobacco withdrawal symptoms	1 y
Lee EJ 2020	109<br>I1:41/1<br>I2:31/1<br>C:33/2	Adult smokers	Auricular acupressure	Auricular acupressure + positive group psychotherapy motivational interview *vs*. Placebo auricular acupressure + positive group psychotherapy motivational interview *vs*. Self-help smoking cessation (6 w)	Change in cigarette smoking; abstinence rate	1 y
Liu TY 2019	301<br>I1:89/16<br>I2:84/20<br>C:87/15	Smokers with syndrome of intermingled phlegm and blood stasis	TCM quit smoking patch	TCM patch + sham nicotine patch *vs*. Sham TCM patch + nicotine patch *vs*. Sham TCM patch + sham nicotine patch (4 w)	Abstinence rate; relapse rate; TCM syndrome efficacy, daily cigarette consumption; expiratory CO content; serum cotinine; FTND; tobacco craving; MNWS	4 w

Ma W 2014	136<br>I:68<br>C:68	Smoker	Acupuncture	Acupuncture *vs*. Placebo (sham ac upuncture) (8w)	Abstinence rate, concentration of serum sialic acid and C-reactive protein	6m
Parker LN 2007	41<br>I1/C1:9/9;<br>I2/C2:11/12	Smoker	Auricular acupoints intradermal needling; auricular acupoints TEAS	Auricular acupoints intradermal ne edling vs auricular acupoints TEA S vs non-specific acupoints stimul ation vs non-specific auricular acu points TEAS (3w)	NA	NA
Qin YB 2020	180<br>I1:53/7<br>I2:57/3<br>C:56/4	Tobacco dependence with phlegm and blood stasis syndrome	Chinese herbal quit smoking Babu Patch; Chinese herbal quit smoking Babu Patch combining acupuncture	Chinese herbal quit smoking Babu Patch vs quit smoking Babu Patc h combining acupuncture vs place bo Babu Patch combining acupunc ture (4 w)	Clinical effectiveness, TCM syndrome score, FTND, MNWS, withdrawal rate, relapse rate	6 m
Sood A 2010	118<br>I1:22/18<br>I2:18/21<br>C:13/26	Adult smokers	TCM extraction	TCM extraction (900mg/d) *vs*. TC M extraction (1800mg/d) *vs*. Place bo (12 w)	7-day point prevalence abstinence, 6-month continuous abstinence	12 w
Steiner RP 1982	32	Adult smokers	Acupuncture	Acupuncture *vs*. Sham acupuncture (NR)	Daily cigarette consumption	NR

Sun X 2009	48<br>I:24/0<br>C:24/0	Adult smokers	Kangyinjian liquid patent	Kangyinjian liquid *vs*. Placebo (3 w)	Abstinence rate, smoking reduction, FTND	12 w
Waite NR 1998	78<br>I:22/18<br>C:21/17	Adult smokers	Auricular electroacupuncture+ Auricular acupressure	Auricular electroacupuncture + aur icular acupressure *vs*. Sham auricu lar electroacupuncture + auricular acupressure (2 w)	Urinary cotinine; abstinence rate	6 m
White AR 1998	76<br>I:17/21<br>C:20/18	Adult smokers	Electroacupuncture	Electroacupuncture *vs*. Sham electr oacupuncture (2 w)	Cessation symptom score, abstinence rate	2 w
LI YK 2010	70<br>I:26/12<br>C:23/9	Adult smoker	Auricular Acupressure	Auricular acupressure vs sham acu pressure (non-specific non-meridia n points) (3w)	FTND, MPSS, daily cigarette consumption, expiratory CO content	NR
Wu TP 2007	118<br>I:48/11<br>C:52/7	Adult smokers	Auricular acupuncture	Auricular acupuncture *vs*. Sham a uricular acupuncture (8 w)	Daily cigarette consumption; nicotine cessation symptom score	6 m

Zhang AL 2013	43<br>I:8/12<br>C:10/13	Adult smokers	Auricular acupressure	Specialized smoking-cessation auri cular acupressure *vs.* Non-specific smoking-cessation auricular acupre ssure (8 w)	7-day point prevalence abstinence, NWS assessment using the Mood and Physical Symptom Scale (MPSS), NRT consumption dosage during the trial, daily cigarette consumption, body weight, quality of life	12 w
Zhao HJ 2018	210<br>I:111/13<br>C:69/7	Adult smokers	TCM patch	TCM application group *vs.* Placeb o application group (4 w)	Cigarette consumption, discomfort, change in appetite, sleep quality	NR
TCM vs. no treatment, 6 trials						
Bai L 2001	80<br>I1:20<br>C1:20<br>I2:20<br>C2:20	Male college smoker	Acupuncture	Acupuncture Mr. Ren's smoking c essation area *vs.* Blank control (2 0 d)	Blood cotinine level	NR
Chen H 2019	180<br>I1:50<br>I2:50<br>I3:50<br>C:30	Adult smokers	Lung clearing and quit smoking tea	Clear lung quit smoking tea *vs.* N o intervention (1 y)	Ventilation function, abstinence rate	NR

Li Y 2009	140<br>I:52/18<br>C:55/15	Adult smokers	Auricular acupressure	Auricular acupressure *vs*. Blank co ntrol (1 m)	Abstinence rate, urinary sulfatide	3 m
Reif MH 1999	20<br>I:3/7<br>C:5/5	Medical school smoker	Massage	Hand/ear massage *vs*. Blank contr ol (1 m)	Anxiety, emotional state, tobacco craving, daily cigarette consumption	NR
Silva RP 2014	30<br>I:21<br>C: 9	Adult smoker	Auriculotherapy	Auriculotherapy vs blank control (auriculotherapy in points consider ed irrelevant to tobacco control) (10w)	NCS, DND, expiratory CO content	1m
Yeh ML2014	112	Adult smokers	Auricular acupressure	Auricular acupressure vs. Interacti ve media vs. Blank control (10 w)	Expiratory CO content; cotinine level; nicotine dependence	NR
TCM vs. Other non-drug therapies, 4 trials						
Chu C 2013	46	Male college smoker	Reinforcing and reducing manipulations of acupoints	Gua sha + magnetic bead auricula r acupressure *vs*. Magnetic bead a uricular acupressure *vs*. Psychologi cal counseling (nr)	Expiratory CO content, FTND, cessation symptom scale, SCL90 scale and depression scale	14 d
Guo B 2020	120<br>I:39/21<br>C:39/21	Adult smokers	Auricular acupressure + 5A	Auricular acupressure + 5a group *vs*. Consultation (4 w)	Abstinence rate, daily cigarette consumption	6 m

He HJ 2019	60<br>I:19/11<br>C:17/13	Psychiatric smoking patient	Fire needle therapy	Fire needle therapy *vs*. Education (3 w)	Change in cigarette consumption, CO degree, abstinence rate, and cessation symptoms	3 w, 8 w
Zhao L 2017	50<br>I:23/4<br>C:21/2	Smoking patients in psychiatric department	Auricular acupressure	Auricular acupressure *vs*. Cognitiv e behavioral therapy (cbt) (8 w)	Abstinence rate, FTND, MNWS, expiratory CO content	24 w
TCM + Other non-drug therapies vs. Other non-drug therapies, 2 trials						
Gong ZY 2008	90	Smoking patients in psychiatric department	Auricular acupressure, TCM alternative cigarette	Primary care *vs*. Primary care + a uricular acupressure *vs*. Primary c are + auricular acupressure + alter native cigarette (herbal) (not repor ted)	Total effective rate, abstinence response	NR
Leung JP 1977	64<br>I:32<br>C:32	Smoker who had smoked ≥ 1 year and was motivated to stop	Auricular acupoints intradermal needling + counseling	Auricular acupoints intradermal ne edling + counseling vs. counseling (2w)	NA	NA
Other comparisons, 3 trials						
Chen JH 2016	80<br>I:40/0<br>C:40/0	Patients with "smoking lung"	Qingfei Huatan Liyan Decoction	Qingfei Huatan Liyan Decoction+ Ceftriaxone sodium vs Ceftriaxone sodium (2 w)	Clinical effectiveness, abstinence rate	6 m

Li Z 2018	286<br>I:124/38<br>C:102/22	Adult smokers	TCM treatment based on syndrome differentiation	Oral antidepressant sertraline + TCM treatment based on syndrome differentiation dialectical treatment *vs*. Non-nicotine replacement drug varenicline lithate (6 m)	Abstinence rate, FTND, and improvement in symptoms	6 m
Zeng XJ 2021	100<br>I:40/10<br>C:39/11	Adult smokers	Catgut-embedding therapy	Catgut-embedding therapy + cognitive behavioral Therapy + Varenicline vs Varenicline (8 w)	Abstinence rate, pulmonary function test, quality of life, patient compliance and satisfaction	NR
Multigroup, 9 trials						
Chai X 2019	500<br>I1:93/7<br>I2:92/8<br>I3:94/6<br>I4:95/5<br>C1:95/5	Adult smokers	Acupuncture, auricular acupuncture, electroacupuncture	Acupuncture *vs*. Auricular acupuncture *vs*. Acupuncture + auricular acupuncture *vs*. Electroacupuncture *vs*. NRT (8 w)	Point prevalence abstinence; continuous abstinence rate, safety indicators; tobacco dependence (FTND, HSI)	16 w
Chapelon FC 1997	998(548/450)<br>I1:272<br>I2:268<br>C1:243<br>C2:213	Adult smokers	Acupuncture	2×2 design with acupuncture and nicotine gum (4 w)	Abstinence rate	4 y

Chandrabhaga SV 2021	150<br>I1:50<br>I2:50<br>C:50	Adult smokers	Laser auricular acupressure	Laser auricular acupressure + Psychological counseling vs Laser auricular acupressure vs Psychological counseling (4 w)	FTND, VAS	1 m
Cottraux JA 1983	558<br>I:108/32<br>C1:105/33<br>C2:105/35<br>C3:118/22	Adult smokers	Acupuncture	Acupuncture *vs*. Behavioral therapy *vs*. Placebo drug *vs*. Blank control (2 w)	Abstinence rate, daily cigarette consumption	13 m
Kuang HJ 2021	90<br>I1:28/2<br>I2:28/2<br>C:29/1	Adult smokers	Auricular acupressure	Auricular acupressure + Transcranial direct current Stimulation (tDCS) vs Auricular acupressure vs Transcranial direct current stimulation (tDCS) (4 w)	Daily cigarette consumption ,FTND, MNWS, QSU, Urine cotinine concentration	4 w
Yavagal PC 2021	56	Young adult smoker aged 18–35	Laser auricular acupuncture	Laser auricular acupuncture vs psychological counselling vs laser auricular acupuncture + psychological counselling (2 w, 4 w)	FTND, urinary cotinine levels, cigarettes/bidis smoked per day	NR

Zeng XJ 2019	120<br>I1:30/10<br>I2:32/8<br>C:31/9	Patients with tobacco dependence	Acupoint catgut embedding therapy	Acupoint catgut embedding therap y + varenicline vs acupoint catgut embedding therapy vs varenicline (12w)	Clinical effectiveness, FTND, HSI, expiratory CO, quality of life	NR
Jiang B 2011	68<br>I1:21<br>I2:23<br>C:24	Military personnel	Auricular acupressure	Psychological intervention *vs.* psyc hological intervention + vareniclin e *vs.* Psychological intervention + auricular acupressure (nr)	Change numbers of cigarette smoking; continuous abstinence; point prevalence abstinence	1, 3, 6 and 12 m
Li LB 2011	300<br>I1:55/2<br>I2:62/1<br>C1:61/1<br>C2:54/1	Adult smokers	Auricular acupoint stimulation	Psychological intervention *vs*. Auri cular acupoint stimulation *vs*. Psyc hological intervention + auricular acupoint stimulation *vs*. Blank con trol group (2 m)	Abstinence rate, daily cigarette consumption	6 m
TCM vs. TCM, 15 trials						
Cai ZB 2022	90<br>I1:29/1<br>I2:29/1<br>I3:30/0	Adult smokers	Auricular acupressure, acupuncture	Auricular acupressure + Acupunctur e vs Auricular acupressure vs Acup uncture (8 w)	Exhaled CO, abstinence rate, FTND, AUTOS, daily cigarette consumption, adverse event	NR

Gu ZR 2007	400<br>I1:100<br>I2:100<br>I3:100<br>I4:100	Adult smokers	TCM acupoint application, odor therapy, laser needling	TCM acupoint patch + odor thera py + laser needling *vs*. TCM acu point patch *vs*. Odor therapy *vs*. Laser needling (nr)	Overall effective rate (smoking urge)	NR
Han Y 2006	42<br>I1:20<br>I2:22	Adult smokers	Auricular acupressure; Auricular acupressure + body acupuncture	Auricular acupressure *vs*. Auricular acupressure + body acupuncture (10 d)	Abstinence rate, over all effective rate	1 m
Liu D 2015	48<br>I1:20/4<br>I2:18/6	Adult smokers	Auricular acupressure; auricular acupressure + body acupuncture	Auricular acupressure *vs*. Auricular acupressure + body acupuncture (1 m)	Cessation rates, FTND, daily cigarette consumption, and Tobacco Dependence Self-Rating Scale	NR
Ji J 2022	220<br>I1:105/5<br>I2:106/4	Adult smokers	Acupuncture	High Frequency Acupuncture vs Low Frequency Acupuncture (8 w)	Abstinence rate, expiratory CO, urinary cotinine levels, FTND, HIS, MNWS, QSU-Brief, adverse event	12 w
Sun CX 2000	60<br>I:9/23<br>C:7/21	Male college smoker	Auricular acupressure	Auricular acupressure + psychological care vs. Auricular acupressure (7 d)	Overall effective rate (degree of reduction in daily smoking), improvement in anxiety and depression	1 m

Yeh ML 2009	79<br>I:39<br>C:40	Adult smokers	Electroacupuncture, ear pressure bean	Electroacupuncture + auricular acu pressure beans *vs*. Sham electroac upuncture + auricular acupressure beans (6 w)	Blood cotinine; expiratory co content; tobacco consumption; abstinence rate	NR
Wan ZX 2021	133<br>I:66/1<br>C:63/3	Smoker who relapses	Auricular acupressure + Cognitive behavioral therapy (CBT)	Auricular acupressure + Cognitive behavioral therapy (CBT) *vs*. auri cular acupressure (8 w)	Relapse rate, FTND, HIS, MNWS, QSU-Brief, expiratory CO	6 m
Wang DF 2021	160<br>I:71/9<br>C:71/9	Smoker willing to quit smoking	Auricular Point Sticking	Auricular point sticking (main acu points + acupoints based on const itution identification) vs Auricular point sticking (main acupoints) (3m)	Clinical effectiveness	NR
Wang WH 2013	60<br>I1:15/15<br>I2:16/14	Adult smokers	Acupuncture; acupuncture + moxibustion	Acupuncture *vs*. Acupuncture + m oxibustion (1 m)	Abstinence rate, overall effective rate	None
Wang XJ 2013	80<br>I1:39/1<br>I2:38/2	Adult smokers	TCM compound intervention (TCM tea, auricular acupressure, acupoint patch)	TCM complex intervention *vs*. TC M tea + multi-enzyme tablets (4 w)	Abstinence rate, overall effective rate, decreased of levels of thiocyanate in morning urine	6 m

Zhou JH 2010	60<br>I1:13/17<br>I2:13/17	Adult smokers	Acupuncture group, electroacupuncture + auricular acupuncture group	Acupuncture group *vs.* Electroacupuncture + auricular group (20 d)	Abstinence rate	6 m
Zhang JM 1989	120<br>I1:60/0<br>I2:60/0	Adult smokers	Body acupoints, auricular acupoints pressed medicine	Acupressure with cowherb seed *vs.* Acupressure with white mustard seed, lycopodium, and soursop (6 d)	Total effective rate (daily smoking reduction)	NR
Zhang QG 2004	60<br>I1:30/0<br>I2::30/0	Adult smokers	Wrist and ankle acupuncture, acupuncture	Wrist and ankle acupuncture *vs.* Conventional acupuncture (4 w)	Over all effective rate (daily smoking reduction)	1 y
Zhang X 2016	60<br>I1:28/2<br>I2:29/1	Smoker with syndrome of liver depression and qi stagnation	Acupuncture	Acupuncture at sub-matrix points of liver meridian *vs.* Conventional acupuncture (2 w)	Overall effectiveness	3 m

Notes: M=male; F=female; y=year; m=month; w=week; d=day; NR=Not reported; NA=Not available; TCM=Traditional Chinese medicine; 5A= "Ask, advise, assess, assist, arrange" intervention; CO=Carbon Monoxide; FTND=Fagerstrom Test of Nicotine Dependence; HSI=Heaviness of smoking index; MNWS=Minnesota Nicotine Withdrawal Symptoms Scale; QSU-Brief= Brief Questionnaire of Smoking Urges; MPSS= Mood and Physical Symptom Scale; BDI=Beck depression inventory; BAI=Beck anxiety inventory; TEAS=Transcutaneous Electrical Acupoint Stimulation; DND=Degree of Nicotine dependence; NCS=Number of cigarettes smoked

Supplementary Table 6 Risk of bias and implementation details of blinding of RCTs of traditional Chinese medicine for smoking cessation

**Study ID**	**Blinding of participant and trial personnel**	**Blinding of outcome assessor**	**Object of blinding**	**Implementation details of blinding**	**Comparison**
**Bier ID 2002**	High	NC	Participant	Fake points: within 5mm from the real acupoint	Acupuncture vs. acupuncture + 5 weeks of smoking cessation education vs. sham acupuncture + 5 weeks of smoking cessation education
**Bilici M 2016**	Low	Low	Participant and outcome assessor	Reduced current level: 10Hz→＜1Hz	Electroacupuncture vs. sham electroacupuncture
**Chae YB 2011**	Low	NC	Participant	In effective quit smoking acupoint, blunt needles	Acupuncture vs. sham acupuncture
**Cai YM 2000**	Low	Low	Participant and outcome assessor	Blindfolded treatment with no radiation in placebo group	Laser needle vs. placebo

**Cai ZB 2022**	High	NC	Participant	NR	Auricular acupressure + Acupuncture vs Auricular acupressure vs Acupuncture
**Clavel FC 1997**	Low	NC	Participant	Two cm away from the investigational acupoints; Placebo gum with the same appearance and taste of nicotine gum	2×2 design, acupuncture and nicotine gum
**Chu C 2013**	High	NC	Participant	NR	Gua Sha + auricular acupressure with magnetic bead vs auricular acupressure with magnetic bead vs psychological counseling

**Chandrabhaga SV 2021**	High	High	No blinding	No blinding	Laser auricular acupressure + Psychological counseling vs Laser auricular acupressure vs Psychological counseling
**Chen SM 2022**	High	Low	Outcome assessor	NR	Transcutaneous electrical acupoint stimulation + Auricular acupressure vs Nicotine patch
**Dai RJ 2022**	NC	NC	Participant	NR	Acupuncture + Nicotine Patch vs Acupuncture + Sham Nicotine Patch vs Sham Acupuncture + Nicotine Patch vs Sham Acupuncture + Sham Nicotine Patch

**Deborah JF 2013**	Low	NC	Participant and trial personnel	Same acupoints, reduced current level from 80Hz to 0Hz with unawareness of personnel in contact with the patients	Auricular needle electrical stimulation vs. sham auricular needle electrical stimulation
**Georgiou AJ 1998**	Low	NC	Participant and trial personnel	The machine in the treatment group was connected; the machine in the control group was not	Electrical stimulation vs. sham electrical stimulation
**Gilbey V 1977**	low risk	unclear	Participants, personnel	NR	Auricular acupoints intradermal needling vs. inactive auricular point ('Kidney') stimulation
**Gillams J 1984**	low risk	unclear	Participants, personnel	NR	Auricular acupoints intradermal needling vs. non-specific acupoints stimulation

**He D 1997**	Low	NC	Participant	Non-smoking-cessation/respiratory acupoints	Specialized smoking-cessation acupoints vs. Non-smoking-cessation acupoints
**He D 2001**	Low	NC	Participant	Non-smoking-cessation/respiratory acupoints	Specialized smoking-cessation acupoints vs. Non-smoking-cessation acupoints
**Huang Y 2012**	NC	NC	Participant	Auricular acupoint: replaced the cowherb seeds with the sponge of the same color and size; body acupoint: rubber ring placed on the acupoint, cut truncated into the length of the needle, so that the appearance is consistent, the tip of the needle touches the epidermis and does not penetrate the skin	Smoke-san acupuncture combined with auricular acupressure vs Smoke-san acupressure combined with auricular acupressure + placebo acupuncture

**Hyun MK 2010**	Low	Low	Participant and outcome assessor	Ineffective smoking-cessation acupoint: 1 cm away from the investigational acupoints, inserted 3mm	Acupuncture vs. sham acupuncture
**Jang SB 2019**	High	Low	Outcome assessor	Outcome accessor was unaware of the grouping	TCM + NRT + consulting vs NRT + consulting
**Ji J 2022**	High	Low	Outcome assessor	NR	High Frequency Acupuncture vs Low Frequency Acupuncture
**Kerr CM 2008**	High	NC	Participants, personnel	In Group C treatments were performed on days 1, 3, 7 and 14 using the sham probe.	Laser acupuncture vs. sham laser acupuncture vs. laser acupuncture or sham laser acupuncture

**Kuang HJ 2021**	High	Low	Outcome assessor	Outcome accessor was unaware of the grouping	Auricular acupressure + Transcranial direct current Stimulation (tDCS) vs Auricular acupressure vs Transcranial direct current stimulation (tDCS)
**Lambert C 2011**	Low	NC	Participant and trial personnel	Transcutaneous electrical acupoint stimulation, real (10mA) vs. placebo substitute (5mA) vs. placebo (0mA), physician unaware of machine settings	Electroacupuncture of different intensities vs. sham electroacupuncture
**Lee EJ 2019**	Low	High	Participant	Auricular acupressure patches without metal protrusions	Auricular point acupressure + group positive psychotherapy motivational interview vs. placebo auricular point acupressure + group positive psychotherapy motivational interview vs. self-help smoking cessation

**Lee EJ 2020**	High	High	Participant and outcome assessor (failed)	Auricular acupressure patches without metal protrusions	Auricular point acupressure + group positive psychotherapy motivational interview vs. placebo auricular point acupressure + group positive psychotherapy motivational interview vs. self-help smoking cessation
**Lee SN 2016**	Low	NC	Participant	Non-smoking-cessation/respiratory acupoints	Auricular acupressure with beans vs placebo
**Leung JP 1991**	High	High	No blinding	No blinding	auricular acupoints intradermal needling + counseling vs. counseling

**Liu TY 2019**	Low	NC	Participant and trial personnel	Identical looking of quit smoking patches (placebo patches)	TCM patch + fake nicotine patch vs fake TCM patch + nicotine patch vs fake TCM patch + fake nicotine patch
**Ma W 2014**	high risk	high risk	Participant	Control Group received sham acupuncture in the eyes, eyebrows, shoulders and knees, none of which were associated with quitting smoking	Acupuncture vs. Placebo (sham acupuncture)
**Parker LN 1977**	low risk	unclear	Participants, personnel	NR	T1: auricular acupoints intradermal needling; T2: auricular acupoints TEAS vs. C1: non-specific acupoints stimulation; C2: non-specific auricular acupoints TEAS

**Qin YB 2020**	low risk	unclear	Participant	Placebo Babu Patch contains no active ingredients and has the same shape, odor and appearance as the treatment group does.	Chinese herbal quit smoking Babu Patch vs quit smoking Babu Patch combining acupuncture vs placebo Babu Patch combining acupuncture
**Steiner RP 1982**	Low	Low	Participant and outcome assessor	Non-smoking-cessation/respiratory acupoints	Specialized smoking-cessation acupoints vs. Non-smoking-cessation acupoints
**Silva RP 2014**	high	high	Participants, personnel	Auriculotherapy in points that have no effect on the focus of research.	Auriculotherapy vs auriculotherapy in points that have no effect on the focus of research
**Sood A 2010**	Low	NC	Participant and trial personnel	Same appearance of drug in treatment group and in placebo group	Chinese herbal extraction (900mg/d) vs. Chinese herbal extraction (1800mg/d) vs. placebo

**Sun X 2009**	Low	NC	Participant and trial personnel	The placebo was prepared by the research team, and was similar with the appearance of the investigational drug	Kangyinjian liquid vs. placebo
**Yavagal PC 2021**	high	low	Outcome assessor	All interventions were done by an investigator who was not blinded to the treatment procedure	laser auricular acupuncture vs. psychological counselling vs. laser auricular acupuncture + psychological counselling
**Yeh ML 2009**	NC	NC	Participant	Five cm away from the investigational acupoints	Electroacupuncture + auricular acupressure with beans vs. sham electroacupuncture + auricular acupressure with beans
**Waite NR 1998**	NC	Low	Participant and outcome assessor	Needle and cowherb seed placed on the skin surface with zero pointer reading	Auricular electroacupuncture + auricular acupressure vs. sham auricular electroacupuncture + auricular acupressure

**Wan ZX 2021**	high	high	No mentioned	NR	Auricular acupressure + Cognitive behavioral therapy (CBT) vs. auricular acupressure
**Wang DF 2021**	high	high	No mentioned	NR	Auricular point sticking (main acupoints + acupoints based on constitution identification) vs Auricular point sticking (main acupoints)
**White AR1998**	Low	Low	Participant and outcome assessor	The needle placed in the mastoid process of temporal bone without acupoints and current reduced from 100Hz to 0	Electroacupuncture vs. sham electroacupuncture
**Wing YK 2010**	high risk	unclear	Participants, personnel	Non-specific non-meridian points were chosen away from those selected for the treatment group	Auricular acupressure vs. sham auricular acupressure

**Wu TP 2007**	Low	NC	Participant	Non-smoking-cessation/respiratory acupoints	Auricular acupuncture vs. sham auricular acupuncture
**Zeng XJ 2019**	High	High	No mentioned	NR	Acupoint catgut embedding therapy + varenicline vs acupoint catgut embedding therapy vs varenicline
**Zeng XJ 2021**	High	NC	No mentioned	NR	Catgut-embedding therapy + cognitive behavioral Therapy + Varenicline vs Varenicline
**Zhang AL 2013**	Low	Low	Participant and outcome assessor	Non-smoking-cessation/respiratory acupoints	Specialized smoking-cessation auricular acupressure vs. non-specialized smoking-cessation auricular acupressure

**Zhang N 2022**	High	NC	No mentioned	NR	Acupuncture + Auricular acupressure vs Nicotine patch
**Zhang X 2016**	NC	NC	Participant	Acupoints different from the treatment group	Acupuncture at sub-matrix points of liver meridian vs. conventional acupuncture
**Zhang Y 2017**	high risk	high risk	No mentioned	NR	Acupuncture+ intradermal needling vs Nicotine gum
**Zhao HJ 2018**	Low	NC	Participant	Identical looking quit smoking patches (placebo patches)	TCM application group vs. placebo application group

Notes: NC=Not Clear; NR=Not Reported; TCM= Traditional Chinese Medicine; NRT= Nicotine Replacement Therapy

Supplementary Table 7 Summary of abstinence rate from meta-analysis of RTCs on smoking cessation in TCM

Study ID	Sample size	Main intervention	Effect size RR (95%)	Outcomes	P-value
**TCM vs. NRT**					
Wang YY 2017	T:100 C:100	Acupuncture	0.84 [0.50, 1.40]	7-day point prevalence abstinence	0.5010
Wang YY 2017	T:100 C:100	Auricular acupuncture	1.00 [0.62, 1.62]	7-day point prevalence abstinence	1.0000
Chen SM 2022	T:100 C:100	Auricular acupuncture	0.89 [0.56, 1.42]	2-month point prevalence abstinence	0.6306
Wu Y 2012	T:30 C:32	Acupuncture	1.14 [0.69, 1.87]	2-month point prevalence abstinence	0.6105
Wu Y 2012	T:30 C:32	Acupuncture	1.14 [0.69, 1.87]	5-month point prevalence abstinence	0.6105
Peng ZJ 2015	T:30 C:30	Acupuncture + auricular acupressure	0.33 [0.01, 7.87]	Less than 7-day continuous abstinence	0.4557
Peng ZJ 2015	T:30 C:30	Acupuncture + auricular acupressure	0.33 [0.04, 3.03]	7-day to 1-month continuous abstinence	0.2963
Zhang N 2022	T:41 C:41	Acupuncture	0.89 [0.38, 2.08]	7-day to 1-month continuous abstinence	0.7852
Wang YY 2017	T:100 C:100	Acupuncture	0.78 [0.56, 1.10]	1 to 6 months continuous abstinence	0.1484

Study ID	Sample size	Main intervention	Effect size RR (95%)	Outcomes	P-value
Wang YY 2017	T:100 C:100	Auricular acupuncture	0.70 [0.49, 0.99]	1 to 6 months continuous abstinence	0.0403
Clavel F 1985	T:224 C:205	Acupuncture	0.86 [0.59, 1.24]	1 to 6 months continuous abstinence	0.4089
Peng ZJ 2015	T:30 C:30	Acupuncture + auricular acupressure	0.75 [0.30, 1.90]	1 to 6 months continuous abstinence	0.5403
Wu Y 2012	T:30 C:32	Acupuncture	1.28 [0.44, 3.76]	1 to 6 months continuous abstinence	0.6527
Li XP 2013	T:33 C:35	TCM tablet	0.61 [0.36, 1.03]	1 to 6 months continuous abstinence	0.0447
Zheng X 2019	T:28 C:27	TCM quit smoking patch	1.04 [0.59, 1.87]	1 to 6 months continuous abstinence	0.8825
Zhang N 2022	T:41 C:41	Acupuncture	1.33 [0.51, 3.50]	1 to 6 months continuous abstinence	0.5564
Chen SM 2022	T:100 C:100	Auricular acupuncture	0.94 [0.65, 1.38]	1 to 6 months continuous abstinence	0.7668
Wang YY 2017	T:100 C:100	Acupuncture	0.30 [0.17, 0.51]	6 to 12 months continuous abstinence	0.0000
Wang YY 2017	T:100 C:100	Auricular acupuncture	0.68 [0.47, 0.99]	6 to 12 months continuous abstinence	0.0382

Study ID	Sample size	Main intervention	Effect size RR (95%)	Outcomes	P-value
Clavel F 1985	T:224 C:205	Acupuncture	0.65 [0.36, 1.17]	More than 12-month continuous abstinence	0.1497
**TCM vs. Varenicline**					
Jiang B 2011	T:23 C:21	Auricular acupressure	1.22 [0.31, 4.82]	1-month point prevalence abstinence	0.7775
Jiang B 2011	T:23 C:21	Auricular acupressure	1.03 [0.49, 2.17]	6-month point prevalence abstinence	0.9438
Jiang B 2011	T:23 C:21	Auricular acupressure	1.83 [0.52, 6.39]	1 to 6 months continuous abstinence	0.3223
Jiang B 2011	T:23 C:21	Auricular acupressure	1.28 [0.48, 3.42]	6 to 12 months continuous abstinence	0.6199
**TCM vs. Bupropion**					
Li HY 2019	T:49 C:48	Acupoint catgut embedding therapy	0.98 [0.61, 1.58]	1-month point prevalence abstinence	0.9322
Li HY 2019	T:49 C:48	Acupoint catgut embedding therapy	0.98 [0.61, 1.58]	1 to 6 months continuous abstinence	0.9322
**TCM + conventional treatment vs. conventional treatment**					

Study ID	Sample size	Main intervention	Effect size RR (95%)	Outcomes	P-value
Li Z 2018	T:162 C:124	TCM symptoms differentiation treatment	0.75 [0.62, 0.90]	7-day to 1-month continuous abstinence	0.0022
Jang SB 2019	T:20 C:21	Body acupuncture + auricular acupuncture + aromatherapy	1.15 [0.67, 1.97]	1 to 6 months continuous abstinence	0.6220
Li Z 2018	T:162 C:124	TCM symptoms differentiation treatment	1.35 [1.15, 1.58]	1 to 6 months continuous abstinence	0.0001
Li Z 2018	T:162 C:124	TCM symptoms differentiation treatment	1.45 [1.23, 1.70]	6 to 12 months continuous abstinence	0.0000
Jang SB 2019	T:20 C:21	Body acupuncture + auricular acupuncture + aromatherapy	1.23 [0.50, 3.02]	3-month point prevalence abstinence	0.6580
Jang SB 2019	T:20 C:21	Body acupuncture + auricular acupuncture + aromatherapy	1.05 [0.56, 1.97]	6-month point prevalence abstinence	0.8788
**TCM vs. Non-drug therapies**					
Zhao L 2017	T:25 C:25	Auricular acupressure	2.00 [0.98, 4.10]	1-month point prevalence abstinence	0.0365
Zhao L 2017	T:25 C:25	Auricular acupressure	3.00 [1.29, 7.00]	2-month point prevalence abstinence	0.0016

Study ID	Sample size	Main intervention	Effect size RR (95%)	Outcomes	P-value
Lee EJ 2020	T:42 C:32	Auricular acupressure	0.57 [0.14, 2.37]	1 to 6 months continuous abstinence	0.4486
Zhao L 2017	T:25 C:25	Auricular acupressure	5.00 [1.22, 20.55]	1 to 6 months continuous abstinence	0.0043
Lee EJ 2019	T:18 C:19	Auricular acupressure	0.35 [0.02, 8.09]	6 to 12 months continuous abstinence	0.4483
Lee EJ 2020	T:42 C:32	Auricular acupressure	0.76 [0.16, 3.53]	6 to 12 months continuous abstinence	0.7316
Cottraux JA 1983	T:140 C:138	Acupuncture	2.27 [1.12, 4.58]	6 to 12 months continuous abstinence	0.0165
Lee EJ 2019	T:18 C:19	Auricular acupressure	4.22 [0.52, 34.28]	More than 12-month continuous abstinence	0.1251
**TCV vs. placebo**					
Wu TP 2007	T:59 C:59	Auricular acupuncture	1.50 [0.45, 5.04]	3-month point prevalence abstinence	0.5078
Sood A 2010	T:40 C:39	TCM extraction 900mg	1.71 [0.54, 5.37]	3-month point prevalence abstinence	0.3485
Sood A 2010	T:39 C:39	TCM extraction 1800mg	1.75 [0.56, 5.50]	3-month point prevalence abstinence	0.3261

Study ID	Sample size	Main intervention	Effect size RR (95%)	Outcomes	P-value
Sun X 2009	T:24 C:24	Kangyinjian liquid	3.00 [0.13, 70.16]	Less than 7-day continuous abstinence	0.4498
White AR 1998	T:38 C:38	Electroacupuncture	0.94 [0.55, 1.61]	7-day to 1-month continuous abstinence	0.8154
Bier ID 2002	T:22 C:42	Acupuncture	0.78 [0.44, 1.39]	7-day to 1-month continuous abstinence	0.3779
Liu TY 2019	T:105 C:102	TCM quit smoking patch	1.98 [1.32, 2.97]	7-day to 1-month continuous abstinence	0.0003
Li Y 2009	T:69 C:67	Auricular acupressure	2.43 [1.15, 5.13]	7-day to 1-month continuous abstinence	0.0115
Waite NR 1998	T:40 C:38	Auricular electroacupuncture + auricular acupressure	2.04 [0.93, 4.44]	7-day to 1-month continuous abstinence	0.0541
Baccetti S 2015	T:149 C:154	Medicated acupuncture， plum-blossom needle or auricular acupuncture	1.28 [0.93, 1.77]	7-day to 1-month continuous abstinence	0.1314
Qin YB 2020	T:60 C:60	TCM BaBu patch	1.68 [1.24, 2.27]	7-day to 1-month continuous abstinence	0.0001
Sun X 2009	T:24 C:24	Kangyinjian liquid	4.00 [0.95, 16.92]	7-day to 1-month continuous abstinence	0.0250

Study ID	Sample size	Main intervention	Effect size RR (95%)	Outcomes	P-value
Cai YM 2000	T:101 C:107	Laser Needle	0.95 [0.59, 1.51]	1 to 6 months continuous abstinence	0.8147
Bier ID 2002	T:16 C:25	Acupuncture	1.56 [0.61, 4.01]	1 to 6 months continuous abstinence	0.3621
Li Y 2009	T:69 C:67	Auricular acupressure	2.36 [1.05, 5.32]	1 to 6 months continuous abstinence	0.0265
Lee EJ 2019	T:18 C:19	Auricular acupressure	0.21 [0.01, 4.11]	1 to 6 months continuous abstinence	0.2024
Lee EJ 2020	T:42 C:32	Auricular acupressure	0.61 [0.27, 1.37]	1 to 6 months continuous abstinence	0.2312
Dai RJ 2022	T:22 C:22	Acupuncture	2.80 [1.22, 6.44]	1 to 6 months continuous abstinence	0.0026
Waite NR 1998	T:40 C:38	Auricular electroacupuncture + auricular acupressure	18.07 [1.09, 300.14]	1 to 6 months continuous abstinence	0.0010
Sun X 2009	T:24 C:24	Kangyinjian liquid	7.00 [0.93, 52.63]	1 to 6 months continuous abstinence	0.0136
Bier ID 2002	T:16 C:25	Acupuncture	1.56 [0.61, 4.01]	6 to 12 months continuous abstinence	0.3621
Lee EJ 2019	T:18 C:19	Auricular acupressure	0.35 [0.02, 8.09]	6 to 12 months continuous abstinence	0.4483

Study ID	Sample size	Main intervention	Effect size RR (95%)	Outcomes	P-value
Lee EJ 2020	T:42 C:32	Auricular acupressure	0.63 [0.21, 1.90]	6 to 12 months continuous abstinence	0.4217
Waite NR 1998	T:40 C:38	Auricular electroacupuncture + auricular acupressure	10.46 [0.60, 183.01]	6 to 12 months continuous abstinence	0.0261
Baccetti S 2015	T:149 C:154	Medicated acupuncture， plum-blossom needle or auricular acupuncture	1.75 [1.17, 2.61]	6 to 12 months continuous abstinence	0.0047
Bier ID 2002	T:15 C:24	Acupuncture	0.53 [0.28, 1.03]	More than 12-month continuous abstinence	0.0233
Lee EJ 2019	T:18 C:19	Auricular acupressure	4.22 [0.52, 34.28]	More than 12-month continuous abstinence	0.1251
Lee EJ 2020	T:42 C:32	Auricular acupressure	0.76 [0.24, 2.41]	More than 12-month continuous abstinence	0.6474
**TCM vs. Blank**					
Chen H 2019	T:75 C:75	Lung clearing and quit smoking tea	1.20 [1.02, 1.42]	More than 12 months continuous abstinence	0.0241

Study ID	Sample size	Main intervention	Effect size RR (95%)	Outcomes	P-value
Cottraux JA 1983	T:140 C:138	Acupuncture	2.27 [1.12, 4.58]	6 to 12 months continuous abstinence	0.0165
**TCM + non-drug therapies vs. Non-drug therapies**					
Li LB 2011	T:57 C:55	Auricular acupoint stimulation	1.93 [0.70, 5.29]	1 to 6 months continuous abstinence	0.1836

Notes: TCM=traditional Chinese medicine; T=Treatment group; C=control group; RR-risk ratio; CI=confidence interval.

Supplementary Table 8 Adverse events reported in RCTs of traditional Chinese medicine for smoking cessation

**Study ID**	**Sample**	**Main Intervention**	**Comparison**	**Safety Event (n)**
Chen SM 2022	200<br>I:99/1<br>C:98/2	Transcutaneous electrical acupoint stimulation + Auricular acupressure	Transcutaneous electrical acupoint stimulation + Auricular acupressure vs Nicotine patch (8 w)	I: headache 1,<br>C: kin allergy 5
Ji J 2022	220<br>I1:105/5<br>I2:106/4	Acupuncture	High Frequency Acupuncture vs Low Frequency Acupuncture (8 w)	Skin hematom 3, skin bruising 3, dizziness due to tension 2, local tingling 1
Fritz DJ 2013	125<br>I:16/48<br>C:14/47	Auricular electrostimulation	Auricular acupressure vs. sham auricular acupressure (5 w)	15 participants (9 in intervention group, 6 in placebo group), only 1 event considered relate to intervention (auricle discomfort, no redness or swelling)
Li HY 2019	100<br>I：41/8<br>C：40/8	Acupoint catgut embedding therapy	Acupoint catgut embedding therapy vs. Bupropion Hydrochloride Sustained-Release Tablets (7 w)	I: local pain 1, local pain, numb, and swell 3, red and swollen skin 2, induration 1<br>C: nausea 7, upset stomach 4, dry mouth 2, insomnia 1

Study ID	Sample	Main Intervention	Comparison	Safety Event (n)
Liu TY 2019	301<br>I1:89/16<br>I2:84/20<br>C:87/15	TCM quit smoking patch	TCM quit smoking patch + sham nicotine patch vs. sham TCM quit smoking patch + nicotine patch vs sham TCM quit smoking patch + sham nicotine patch (4 w)	26 participants. 11 patients (3 in the experimental group, 6 in the positive drug group, and 2 in placebo group) quit due to skin allergy (rash at the patch spots), all symptoms disappeared after 1-week follow-up; 8 participants (dizziness and headache) and 3 participants (palpitation) were related to the adverse effects of nicotine patches in positive drug group; 1 participant (palpitation) indefinitely related to TCM patch in experimental group; 3 participants (gastrointestinal discomfort, 1 in each of the 3 groups) unrelated to the TCM patches
Qin YB 2020	180<br>I1:53/7<br>I2:57/3<br>C:56/4	Chinese herbal quit smoking Babu Patch	Chinese herbal quit smoking Babu Patch vs. quit smoking Babu Patch combining acupuncture vs placebo Babu Patch combining acupuncture (4 w)	Among the three groups, 4 patients appeared the symptom of skin allergy for longer than half of the treatment course, which disappeared when the treatment stopped.
Sood A 2010	118<br>I1：22/18<br>I2：18/21<br>C:13/26	TCM extraction	TCM extraction (900 mg/d) vs. TCM extraction (1800 mg/d) vs. placebo (12 w)	I1: insomnia 4, dizziness 1, easy to wake up at night 2, clear dreams 1<br>I2: insomnia 1, dizziness 1, headache 1, clear dreams 1<br>C: insomnia 1, dizziness 1, headache 1

Study ID	Sample	Main Intervention	Comparison	Safety Event (n)
Wang YY 2017	300<br>I1：100<br>I2：100<br>C：100	Acupuncture, auricular acupressure	Acupuncture vs. auricular acupressure vs. NRT (8 w)	I1: vertigo 1, hematoma 1<br>I2: None<br>C: local skin irritation 2
White AR 2007	23<br>I1：4/2<br>I2：1/5<br>C:0/7	Auricular acupressure	Binaural acupuncture beads + NRT vs. monaural acupuncture beads + NRT vs. NRT (4 w)	I1: Discomfort 1
Wu TP 2007	118<br>I: 48/11<br>C: 52/7	Auricular acupuncture	Auricular acupuncture vs. sham auricular acupuncture (8 w)	I: small amount of bleeding 2, dizziness 1, nausea 1, residual stabbing sensation 13, pressure pain 23, slight infection 1<br>C: small amount of bleeding 1, dizziness 3, nausea 1, residual stabbing sensation 11, pressure pain 27
Zhang AL 2013	43<br>I：8/12<br>C:10/13	Auricular acupressure	Specialized smoking-cessation auricular acupressure vs. non-specialized smoking-cessation auricular acupressure (8 w)	Mild to moderate ear discomfort<br>I: 1<br>C: 5
Zhang N 2022	82<br>I:36/5<br>C:37/4	Acupuncture + Auricular acupressure	Acupuncture + Auricular acupressure vs Nicotine patch (8 w)	I: fatigue 1;<br>C: skin allergy 4, dizziness and chest tightness 3, fatigue 2

Notes: TCM=Traditional Chinese Medicine; I=Intervention group; C=Control group; W=Week; NRT=Nicotine Replacement Therapy

© 2023 Lu CL. et al.
